# Cleavage of Phosphorothioated DNA and Methylated DNA by the Type IV Restriction Endonuclease ScoMcrA

**DOI:** 10.1371/journal.pgen.1001253

**Published:** 2010-12-23

**Authors:** Guang Liu, Hong-Yu Ou, Tao Wang, Li Li, Huarong Tan, Xiufen Zhou, Kumar Rajakumar, Zixin Deng, Xinyi He

**Affiliations:** 1 Laboratory of Microbial Metabolism and School of Life Science and Biotechnology, Shanghai Jiao Tong University, Shanghai, China; 2 State Key Laboratory of Microbial Resources, Institute of Microbiology, Chinese Academy of Sciences, Beijing, China; 3 Department of Infection, Immunity, and Inflammation, Leicester Medical School, University of Leicester, Leicester, United Kingdom; 4 Department of Clinical Microbiology, University Hospitals of Leicester National Health Service Trust, Leicester, United Kingdom; Universidad de Sevilla, Spain

## Abstract

Many taxonomically diverse prokaryotes enzymatically modify their DNA by replacing a non-bridging oxygen with a sulfur atom at specific sequences. The biological implications of this DNA S-modification (phosphorothioation) were unknown. We observed that simultaneous expression of the *dndA-E* gene cluster from *Streptomyces lividans* 66, which is responsible for the DNA S-modification, and the putative *Streptomyces coelicolor* A(3)2 Type IV methyl-dependent restriction endonuclease ScoA3McrA (Sco4631) leads to cell death in the same host. A His-tagged derivative of ScoA3McrA cleaved S-modified DNA and also Dcm-methylated DNA *in vitro* near the respective modification sites. Double-strand cleavage occurred 16–28 nucleotides away from the phosphorothioate links. DNase I footprinting demonstrated binding of ScoA3McrA to the Dcm methylation site, but no clear binding could be detected at the S-modified site under cleavage conditions. This is the first report of *in vitro* endonuclease activity of a McrA homologue and also the first demonstration of an enzyme that specifically cleaves S-modified DNA.

## Author Summary

Bacteria frequently exchange genetic information among themselves. DNA from one species can be transferred efficiently to unrelated microbes. Bacteria have developed systems that restrict gene transfer. Many restriction systems recognize and destroy foreign DNA entering the cells, but there are also enzymes inducing suicide of cells that have been invaded by foreign genes that modify the host DNA. We describe a restriction endonuclease from an antibiotic-producing soil bacterium that cuts foreign methylated DNA and also foreign DNA containing sulfur. DNA sulfur modification occurs in diverse medically or industrially important microbes and has been shown to prevent cleavage of DNA. The most similar enzyme in the databases is the putative restriction endonuclease McrA from *Escherichia coli* which has not been observed to cleave DNA in a test tube. Our endonuclease showed no activity with magnesium, but it cleaved DNA in the presence of manganese ions. Therefore, we present two novelties: an unusual restriction endonuclease that cleaves sulfur-modified DNA and conditions that allow the study of the enzyme in a test tube.

## Introduction

The sequence of the DNA bases contains the genetic information that is copied with great accuracy and inherited by successive generations. DNA may also carry so called epigenetic modifications that are added by host enzymes after replication. These modifications are lost or changed after transfer of the DNA to a new host by conjugation, transformation or transfection. Epigenetic modifications can also change in response to changing environmental conditions, and they are generally important for eukaryotic gene regulation including carcinogenesis [1].

In bacteria, epigenetic modifications have more specialized roles including the protection of self DNA against restriction endonucleases (REases) which cleave foreign, differently modified DNA. Potential invaders, such as bacteriophages, have developed DNA modifications and other measures to overcome these restriction barriers, and bacteria have evolved to restrict even the modified foreign DNA. One such strategy is for bacteria to restrict methylated DNA [2].

There are many methyl-specific REases. Some cleave DNA at specific methylated recognition sequences. Others make contact with their target DNA at a specific methylated recognition sequence, and then move along the DNA before cleaving at an undefined site. These Type IV methyl-specific REases are very diverse in their amino acid (aa) sequences, and they may consist of one or several peptides [3].

There are about 1303 putative Type IV REases in REBASE (http://rebase.neb.com), but only 3 have been biochemically characterized, whereas others are predicted based on bioinformatic analysis of DNA sequences. Quite unusually, some Type IV REases recognize methylated and also hydroxymethylated or glucosyl-hydroxymethylated DNA [4], [5].

We have discovered a Type IV REase from a bacterium of the genus *Streptomyces* that cleaves methylated and phosphorothioated (S-modified) DNA that has a non-bridging oxygen of the phosphate group in the DNA backbone replaced by sulfur. The DNA S-modification is sequence specific, and a *dnd* (**DN**A **d**egradation) gene cluster encoding four or five proteins is responsible for the S-modification [6], [7].

Streptomycetes are filamentous soil bacteria that produce many chemically diverse antibiotics. The 8.7 Mb linear genome of *Streptomyces coelicolor* A3(2) has been fully sequenced and annotated [8]. *S. coelicolor* contains at least four methyl-specific restriction endonucleases that restrict (reduce or prevent) the introduction of methylated DNA e.g. from Dam^+^ Dcm^+^ Hsd^+^ *E. coli* K-12 strains [9]. Therefore, DNA is generally passaged through a non-methylating *dam dcm hsd E. coli* host before introduction into *S. coelicolor* [10], [11].

Alternatively, the less restricting *Streptomyces lividans* 66 has been used as a recipient for methylated DNA from *E. coli* K. The genes of *S. lividans* and *S. coelicolor* are very similar and highly syntenous except for several genomic islands (GIs), some of which are mobile elements [12], [13].

**Figure 1:**
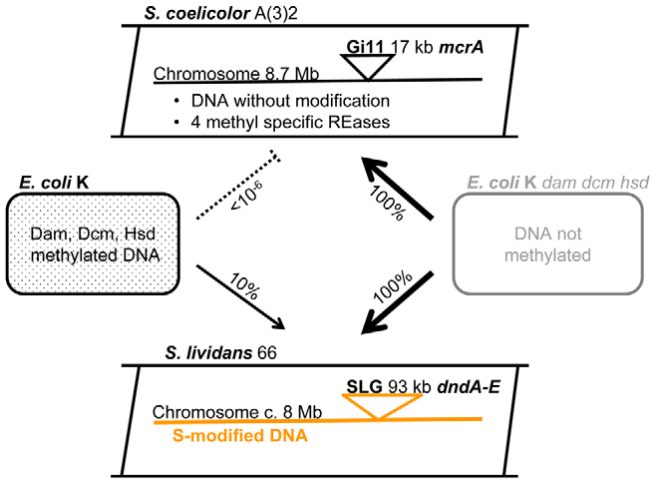
*Streptomyces* and *E. coli* strains used as sources of recombinant DNA. Gi11 ( = SLP1) and SLG are genomic islands (DNA inserts) which are unique to *S. coelicolor* A(3)2 and *S. lividans 66*, respectively. The numbers on the arrows between the *E. coli* and *Streptomyces* cartoons indicate the approximate relative efficiency of plasmid transfer by protoplast transformation as reported by Gonzalez-Ceron et al. (2009). McrA (ScoA3McrA) is remotely similar to the Type IV restriction endonucleases EcoKMcrA and has been shown to restrict methylated DNA *in vivo* in *Stretpomyces* [9]. Dam, Dcm and Hsd are sequence-specific DNA methylases of *E. coli*. *dndA-E* is a gene cluster of *S. lividans* responsible for the sequence-specific S-modification in this strain (only c. 1 in 6000 bp contain sulfur; *dnd* stands for DNA degradation during electrophoresis).

One of these methyl-specific endonucleases, ScoA3McrA (Sco4631) [4] is the focus of this report. As shown schematically in Figure 1, ScoA3McrA is located on the 17.1 kb mobile element Gi11, which is also known as the conjugative and integrative plasmid SLP1 [14].

The Type IV REase EcoKMcrA restricts DNA methylated at the sequences C5mCGG and has been shown to bind to this sequence, but *in vitro* DNA cleavage has not been observed [15]–[17]. Sequence comparison and phylogenetic analysis showed that the ScoA3 and EcoK McrA proteins have very little aa sequence similarities apart from the region surrounding the conserved HNH motif (Figure S7B).

About one in 6000 phosphate groups of the backbone of *S. lividans* genomic DNA contain sulfur instead of a non-bridging oxygen at specific sequences [7], [18]. The stereospecific S-modification (phosphorothioation) renders purified DNA susceptible to oxidative double-strand cleavage by Tris peracid which is generated at the anode during electrophoresis [19]. Five genes, *dndA-E* located on the apparently non-transmissible 93 kb SLG genomic island [13], are involved in DNA S-modification. Evidence for DNA S-modification has been discovered in several *Streptomyces* species, and in phylogenetically diverse prokaryotes including several economically important species [13], [20].

The DNA S-modification of *S. lividans* does not seem to be accompanied by a cognate restriction endonuclease that could defend the strain against the invasion of bacteriophages that lack this modification, and *S. lividans* has traditionally been used as a permissive host for the isolation of many *Streptomyces* phages [11].

In this report we show that S-modified DNA (as well as methylated DNA) is restricted *in vivo* by the *S. coelicolor* methyl-specific endonucleases ScoA3McrA. We also report site-specific *in vitro* cleavage of S-modified DNA by the purified enzyme. This is the first indication of a biological role of DNA S-modification, and also the first report of *in vitro* DNA cleavage by a McrA homologue.

## Results

### Failure to introduce the DNA sulfur modification gene cluster into *S. coelicolor*

**Figure 2:**
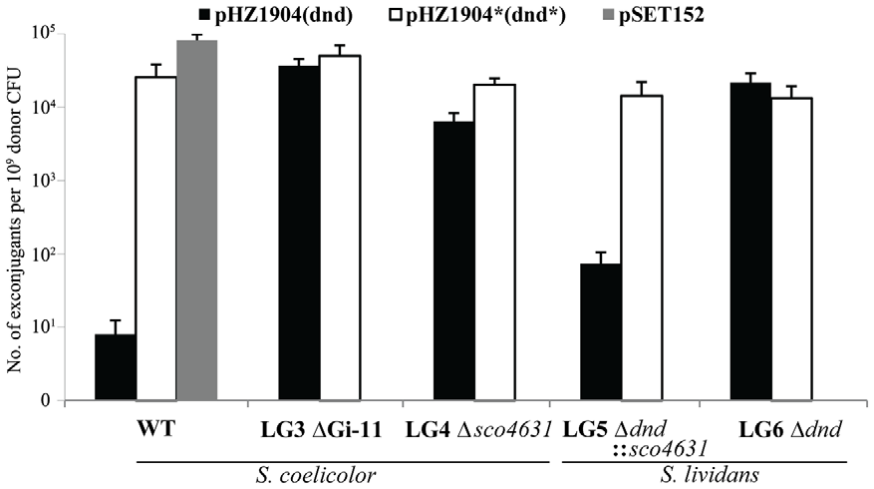
Restriction of plasmids expressing the *dndA-E* gene cluster by *Streptomyces* strains containing *sco4631*. Graph showing the number of apramycin resistant *S. coelicolor* (1–3) and *S. lividans* (4 and 5) exconjugants that were obtained in matings with *E. coli* ET12567/pUZ8002 containing mobilizable (*oriT*) plasmids. WT, plasmid-free, wild-type *S. coelicolor* M145 strain; LG3, *S. coelicolor* mutant lacking the entire genomic island Gi11 ( =  SLP1) which includes *sco4631*; LG4, *S. coelicolor* mutant from which only *sco4631* was deleted; LG5, *S. lividans* derivative which lacks the *dnd* gene cluster and contains a cloned copy of the *S. coelicolor* gene *sco4631*; LG6 *S. lividans* derivative which lacks the *dnd* gene cluster. The graph shows that pHZ1904 which contains the *dndA-E* gene cluster (causes DNA S-modification; black bars), is specifically restricted by the wild type *S. coelicolor* strain. pHZ1904* (white bars), which differs from pHZ1904 by an inactivating frame shift point mutation in *dndE*, was not restricted. The cloning vector pSET152 without insert (grey bar) served as an additional, not restricted control.

The *dndA-E* gene cluster of *S. lividans* is responsible for the DNA S-modification (phosphorothioation) of this strain [6]. We wanted to express this gene cluster in *S. coelicolor* which is closely related to *S. lividans* but lacks these particular genes. In order to generate a stable recombinant *S. coelicolor* strain, we cloned the *dndA-E* gene cluster into the φC31-derived integrative vector pSET152 which has an apramycin resistance marker for selection, and an origin of transfer for highly efficient conjugative transfer from *E. coli* ET12567/pUZ8002 (*dam dcm hsd tra^+^*) to *S. coelicolor* M145. The plasmid vector without cloned DNA (pSET152) produced as expected c. 10^5^ apramycin resistant *S. coelicolor* exconjugants per experiment (one Petri dish). Unexpectedly, pSET152::*dndA-E* (pHZ1904) produced only an average of nine apramycin resistant exconjugants per Petri dish (Figure 2, Panel 1).

These apramycin resistant pSET152::*dndA-E* exconjugants were expected to express the *dnd* genes and generate S-modified *S. coelicolor* DNA that is visibly degraded during agarose gel electrophoresis (methods used in [21]). Only 49 out of 100 exconjugants showed the expected DNA degradation (Dnd^+^ phenotype; Dnd stands for DNA degradation). The remaining 51 contained stable, S-free DNA (Dnd^−^ phenotype, Figure S1A).

The *dndA-E* gene cluster of three Dnd^−^ exconjugants was analysed and each contained a different mutation that was likely to abolish the S-modification activity (Figure S1B).

One of these plasmids, pHZ1904* contained the entire *dndA-E* gene cluster with a single base insertion (frameshift mutation) in *dndE* which is required for DNA S-modification (Figure S1B). pHZ1904* was excised in circular form from *S. coelicolor* and introduced into non-methylating *E. coli* ET12567/pUZ8002. In interspecific matings pHZ1904* produced about 3×10^4^ apramycin resistant *S. coelicolor* M145 exconjugants, almost as many as pSET152 without cloned DNA (Figure 2, Panel 1).

We speculated that S-modification of *S. coelicolor* DNA by the intact *dndA-E* gene cluster was not tolerated because of a hypothetical *S. coelicolor* endonuclease that restricts S-modified DNA. The above 51 Dnd^−^ exconjugants would therefore all contain a mutant, inactive *dndA-E* gene cluster, and the 49 S-modified (Dnd^+^) exconjugants may have kept the *dndA-E* gene cluster intact but may have lost the hypothetical *S. coelicolor* sulfur-specific REase.

We therefore set out to cure the integrated pSET152::*dndA-E* from one of the 49 apramycin resistant Dnd^+^ *S. coelicolor* exconjugants and expected that the resulting strain would be a mutant that allows the reintroduction of fresh pSET152::*dndA-E* at high frequency.

Unfortunately, we could not detect spontaneously apramycin sensitive derivatives of *S. coelicolor* pSET152::*dndA-E*. Instead, we cloned the *dndA-E* cluster into the highly unstable autonomously replicating *Streptomyces* plasmid pJTU412 [22] to generate pJTU1651. Introducing pJTU1651 into *S. coelicolor* M145 by conjugation from *E. coli* produced thiostrepton resistant Dnd^+^ exconjugants. One of these was randomly selected and plated on non-selective medium to allow loss of pJTU1651. LG3, one of the cured (thiostrepton sensitive, Dnd^−^) *S. coelicolor* gave c. 5×10^4^ apramycin resistant exconjugants both with pHZ1904 and with pHZ1904* (Figure 2, Panel 2). As expected, the pHZ1904 exconjugants were Dnd^+^.

These results proved that the process of introducing the *dndA-E* gene cluster into wild-type *S. coelicolor* selected for rare mutant derivatives that no longer restricted the establishment of this gene cluster.

### Identification of the endonuclease gene that prevented the establishment of the *dndA-E*–expressing plasmid

Several putative endonucleases genes had been identified in the *S. coelicolor* genome sequence [12]. The identification of the correct one was aided by the availability of the complete genome sequence of *Streptomyces avermitilis* which contains *dndA-E* homologues and produces S-modified DNA like *S. lividans* [23]. We were thus searching for a putative endonucleases gene of *S. coelicolor* that does not have a counterpart in *S. avermitilis* and found Sco4631 which is encoded by the genomic island Gi11 (also known as SLP1) and known to be absent from *S. lividans* [23]. Sco4631 was shown to restrict methylated DNA in vivo in Streptomyces [9] and is listed in REBASE as ScoA3McrA because it has limited similarity (37% identity including a HNH endonucleases motif in a 91 aa overlap) to EcoKMcrA from E. coli which restricts methylated and hydroxymethylated DNA in vivo, but has not been demonstrated to cleave DNA *in vitro* [4].

The following two experiments tested whether Sco4631 (ScoA3McrA) was indeed the gene that prevented the establishment of pHZ1904 in *S. coelicolor*: single crossover gene disruption using an internal fragment of *sco4631* on a suicide vector plasmid (thiostrepton resistance) was used to produce *S. coelicolor* LG4 (Figure S2). Both pHZ1904 (*dndA-E^+^*) and pHZ1904* (*dndE* ^-^) were introduced by conjugation from *E. coli* ET12567/pUZ8002 with equal high frequency into strain LG4 (c. 10^4^ apramycin-resistant exconjugants per plate, Figure 2, Panel 3).

In addition, *sco4631* including the upstream promoter was cloned into the pSAM2-derived integrating vector pPM927, resulting in pJTU1654, and introduced into HXY16, a *S. lividans* derivative that does not S-modify its DNA because it lacks the entire SLG genomic island including *dndA-E*. The resulting strain, LG5, gave only c. 100 apramycin resistant exconjugants with pHZ1904 but c. 10^4^ exconjugants with pHZ1904* (Figure 2, Panel 4), while LG6, a control strain containing pPM927 without the cloned *sco4631* gene, accepted both pHZ1904 and 1904* with equal high frequency (Figure 2, Panel 5).

These results proved that Sco4631/ScoA3McrA without the need for any other gene on Gi11 (SLP1) restricted the establishment of pHZ1904 which confers S-modification on its host.

### Introduction of *dndA-E* into *S. coelicolor* resulted in the loss of the entire SLP1 sequence

Since Sco4631 is encoded by Gi11, which is also known as the 17.1 kb conjugative plasmid SLP1, we wondered whether the above 49 Dnd^+^ pHZ1904 exconjugants might have lost the entire SLP1 sequence. PCR amplification of LG3 and ten similar exconjugants using outside flanking primers showed the same band. Sequencing of the PCR product of LG3 showed the entire Gi11 sequence was excised precisely, restoring the *tRNA^Tyr^* sequence into which SLP1 had originally inserted (Figure S3A).

This was again consistent with the hypothesis that Sco4631 was responsible for restricting the establishment of pHZ1904 in *S. coelicolor*, and it demonstrated that SLP1 can be lost spontaneously from *S. coelicolor* (Figure S3A). This has not been observed before because SLP1 is a highly efficient conjugative plasmid that would immediately reinfect cured strains in the absence of the *dndA-E* genes.

### Expression of *sco4631* in *S. lividans* resulted in the loss of the genomic island that contains the *dndA-E* gene cluster

pJTU1654 (pPM927 derivative expressing *sco4631*, see above) was introduced by conjugation into wild type *S. lividans* 1326 and into *S. lividans* HXY16 (lacks SLG including dnd*A-E*). A high frequency of 3×10^4^ thiostrepton exconjugants were obtained per plate with S. lividans HXY16, and 300-fold fewer exconjugants were obtained with wild type (Dnd^+^) *S. lividans* 1326. The control pPM927 without cloned DNA transformed both strains with equal frequency (Figure S4).

Ten randomly selected, independent exconjugants were examined using PCR amplification and sequencing of the amplified product. All of them had suffered a precise deletion of the entire 93 kb *S. lividans* SLG genomic island that contains the *dndA-E* gene cluster (Figure S3B). These results again support the hypothesis that *sco4631* and the *dnd* gene cluster are unable to coexist in the same host.

### Cloning and expression of *sco4631* and its mutant derivative in *E. coli*

The coding sequence of *sco4631* including the stop codon was cloned into the expression vector pET28a, generating pJTU1655 for producing amino-terminally His6-tagged Sco4631. A similar plasmid, pSco4631H508A, was constructed that contains an inactive mutant protein because the first histidine residue of the conserved motif (H_508_-N_521_-H_529_) was changed to alanine. The two plasmids were introduced into *E. coli* BL21 (DE3)/pLysE and His_6_-Sco4631 and its mutant derivative were overexpressed for 5 h at 30°C. About 70% percent of the overexpressed protein was soluble in the supernatants and 30% was in the precipitates (Figure S5). The soluble proteins were purified using a Ni affinity column and stored at −20°C in Tris-Cl buffer pH 8.0 containing 50% glycerol.

### His-tagged Sco4631 cleaves Dcm methylated DNA near the site of methylation

In the course of these experiments we noticed that the pET28a derivative containing the cloned *sco4631* gene could not be transformed into Dam^+^ Dcm^+^ *E. coli* DH10B but the plasmid was stably maintained in the Dcm^-^ *E. coli* BL21(DE3)/pLysE expression host and also in the non-methylating *E. coli* ET12567 (see Table S1).

This gave us the idea to test the *in vitro* DNA cleaving activity using as a substrate *dcm* methylated (Cm5CWGG) EcoRV pre-linearized pOJ260 DNA isolated from *E. coli* DH10B.

**Figure 3:**
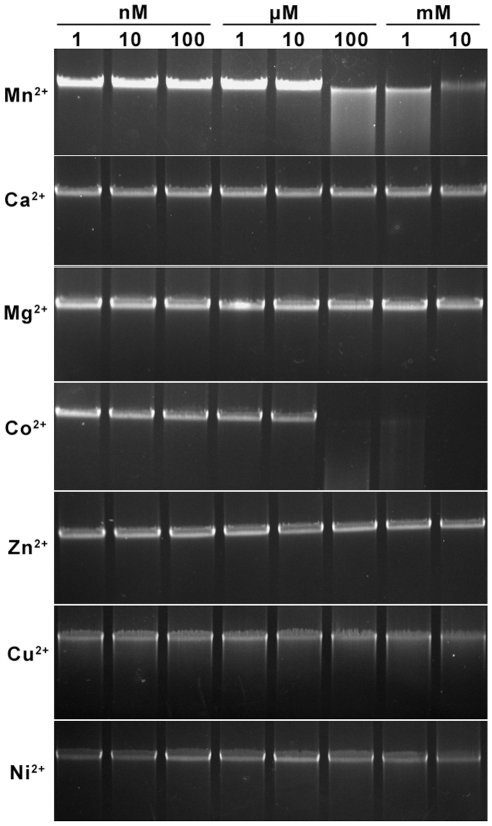
Divalent cation ion requirements analysis for the cleavage of *S. lividans* 1326 total DNA by Sco4631. Total *S. lividans* 1326 DNA (100 ng) prepared by the Kirby mix procedure [11] was used as a substrate to react with 500 ng purified His_6_-Sco4631 protein at 30°C for 5 min in a total reaction volume of 20 µl. The metal ions added to the reaction buffer are indicated to the left, and the concentrations are above the lanes. The reactions were stopped by adding loading buffer containing 1% SDS, 50% glycerol and 0.05% bromophenol blue (Takara). After the reaction, the DNA samples were examined by 0.75% agarose gel electrophoresis. The gel was run at 5 V/cm for 30 min and visualized by staining with ethidium bromide. The strong bands are high molecular weight DNA; the smear observed with Mn^2+^ and Co^2+^ concentrations ≥100 µM indicates DNA cleavage by Sco4631.

No DNA cleavage was observed using standard restriction NEB buffers 1–4 without and with BSA or ATP. Endonuclease activity was, however, observed when Mn^2+^ or Co^2+^ was included in the reaction buffer (Figure 3). Optimal cleavage with minimal unspecific star activity was achieved at 30°C for 5 min using 20 mM Tris-Cl pH 9.0, 50 mM NaCl, 1 mM Mn^2+^.

**Figure 4:**
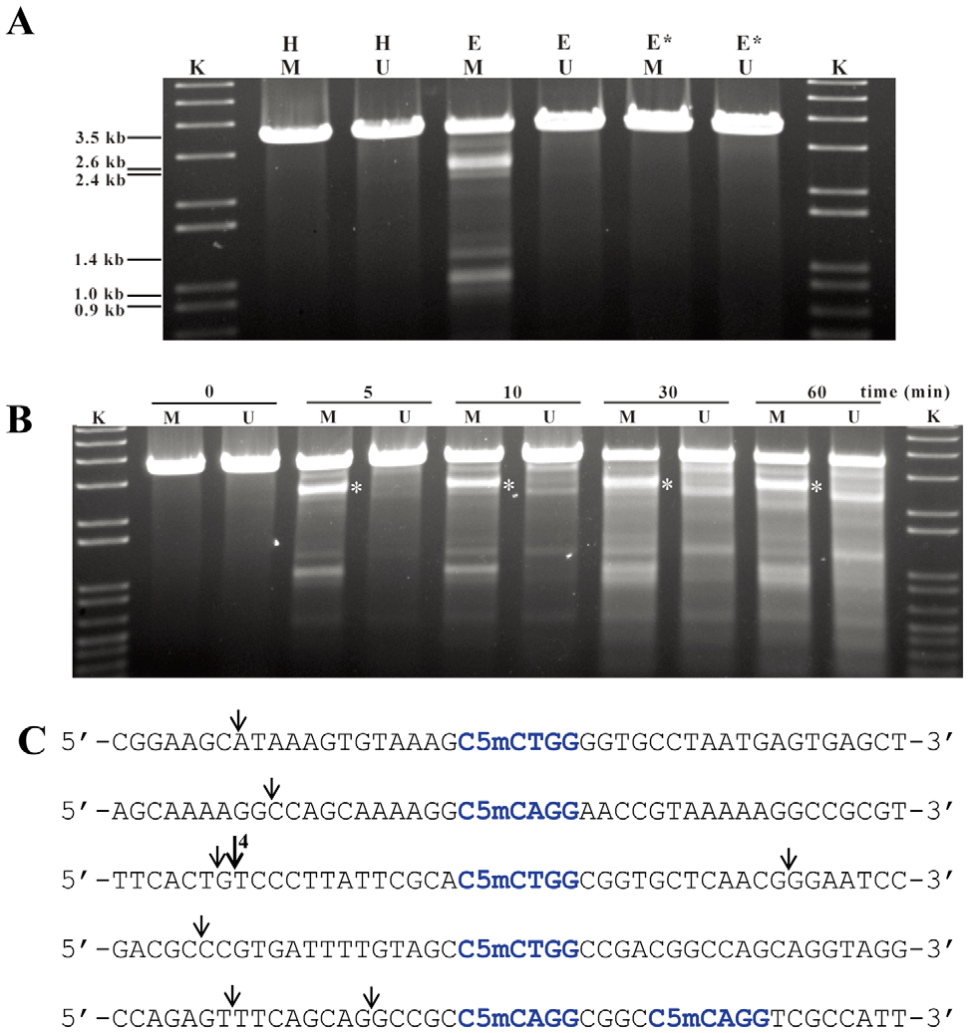
Sco4631-mediated *in vitro* cleavage of Dcm-methylated plasmid DNA. A. Agarose gel showing that DNA cleavage by His_6_-Sco4631 requires DNA methylation. The ethidium bromide-stained agarose gel shows EcoRV linearized pOJ260 DNA treated as indicated above the lanes. Incubation was for 5 min at 30°C in buffer containing 1 mM Mn^2+^. H, heat inactivated His_6_-Sco4631 used as a control; E (Enzyme) and E*, His_6_-Sco4631 and inactive His_6_-Sco4631(H508A), respectively, purified from *E. coli* BL21(DE3); K, kb ladder DNA standard; M (methylated), indicates Dcm methylated pOJ260 that was prepared from Dam^+^ Dcm^+^ host *E. coli* DH10B. U (unmethylated), indicates pOJ260 that was isolated from non-methylating *E. coli* ET12567. The 0.9–2.6 kb bands generated by Sco4631 digestion of methylated DNA are relatively faint, indicating partial digestion. B. Prolonged incubation with His_6_-Sco4631 causes non-specific DNA degradation, also of unmethylated DNA. EcoRV linearized pOJ260 DNA (like in panel A) was incubated for 5–60 min as indicated above the gel. M, methylated DNA; U, unmethylated DNA. C. Sequences of the cleavage sites including the nearby Dcm methylation sites (C5mCWGG, shown in blue). Vertical arrows point to the cleaved bonds, most cuts were observed once except for the cut marked by a larger arrow that was observed in four independent samples. Note, there are four additional C5mCWGG sequences in pOJ260 (not shown) where no cleavage was observed.

Under optimal conditions, about 25% of 100 ng pOJ260 was cleaved and produced at least five bands of differing intensity, indicative of a partial digest (Figure 4A). The precise position of eight cleavage sites was determined by blunt-end ligating the digested pOJ260 DNA to a DNA fragment containing the bla gene conferring ampicillin resistance (see Materials and Methods). The cleavage/ligation sites were sequenced using PCR primers SeqF and SeqR reading away from the ends of the bla fragment. Each of eight cleavage sites was between 12 and 16 bp away from a C5mCWGG Dcm methylation site. pOJ260 has ten Dcm methylation sites and six of them had nearby cut sites (Figure 4C).

These results were consistent with our expectation that Sco4631 is a Type IV restriction endonuclease that cleaves near rather than precisely at its methylated DNA recognition site. (Note that EcoKMcrA does not restrict Dcm-methylated DNA.)

The reaction buffer used in the above experiment resembles buffers that induce star activity (reduction of sequence specificity) in many Type II REases [24], [25]. It might thus be that not all Dcm methylated sites are cleaved in vivo by Sco4631. The fact that cutting near one of the sites was observed six times independently suggested that the DNA recognition sequence of Sco4631 may extend beyond the sequence C5mCWGG, or that Dcm methylation of the plasmid was incomplete as was observed by H. ZHOU (in preparation). Also, the in vivo results of Gonzalez-Ceron et al. [9] showed that Sco4631 restricts DNA containing other DNA methylations.

### His-tagged Sco4631 protein binds and cleaves *in vitro* a synthetic oligonucleotide containing a single Dcm-methylated site

**Figure 5:**
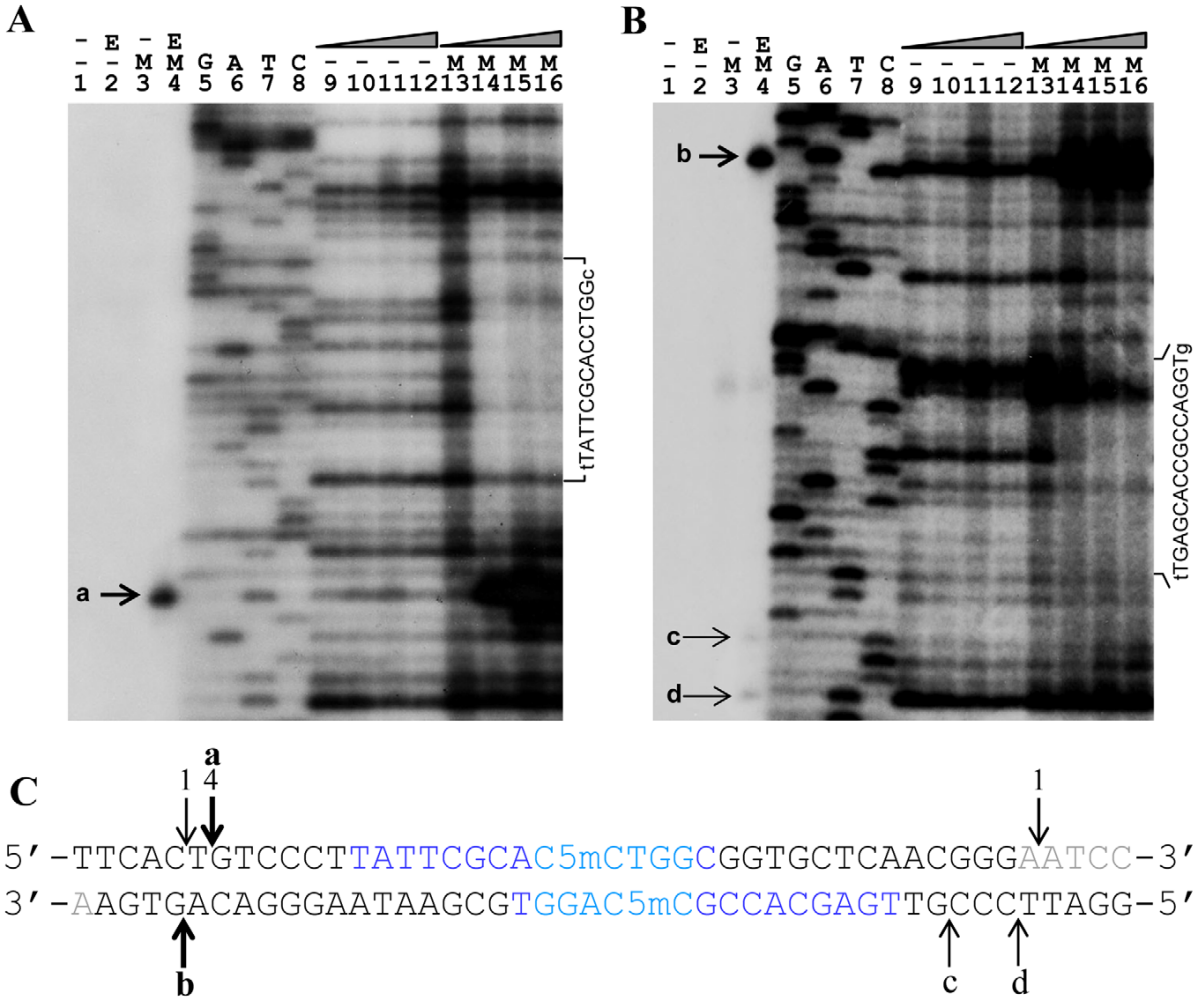
*In vitro* DNase I protection and cleavage of a double-stranded 164 bp Dcm-methylated synthetic oligonucleotide by purified His_6_-Sco4631. A. Analysis of 5′ labeled top strand DNA, and B, 5′ labeled bottom strand DNA. Bands a-d on these autoradiographs represent cleavage of the DNA strands by His_6_-tagged Sco4631. Lanes 1–3 are controls lacking either the enzyme (E) or the Dcm methylation (M). The samples in lanes 4 contained both methylated DNA and active His_6_-Sco4631. Lanes 5–8 are sequencing ladders. Lanes 9–12 are controls containing unmethylated DNA and increasing amounts of active His_6_-Sco4631. Lanes 13–16 contained methylated DNA and increasing amounts of the active His_6_-Sco4631. Lanes 9 and 13 contained no enzyme, and lanes 10–12 and lanes 14–16 contained 1.1, 4.5 and 18 µM enzyme, respectively. The vertical sequence to the right of each gel picture indicates the DNA regions that were partially protected from cleavage by DNase I. The horizontal lines point to bases that are not protected and shown in lower case letters. (Please note, panel A shows the correct sequence of the top strand, but in panel B there is a compression of the sequence with the bold bases in the sequence CCAGGTGCGAATAAG not visible.) C. Central sequence of the 164 bp double-stranded oligonucleotide containing the Dcm methylated sequence C5mCWGG (dark blue) and additional sequences protected against DNase I activity (light blue). The fat vertical arrows labeled a and b are the major cut sites, and the thin arrows labeled c and d are minor cut sites indicated in the gels A and B. Arrows with numbers indicate cut sites that were identified by cloning and sequencing (see Figure 3). Bases printed light grey are not visible on the gel sections shown.

The above results were consistent with the *in vivo* observations suggesting that Sco4631 is a methyl-directed REase, but we could not be absolutely sure that *E. coli* DH10B had not added an unknown, additional modification to pOJ260 that was necessary for target selection by Sco4631. To test whether indeed the Dcm methylation alone was sufficient for cleavage by Sco4631, we synthesized two 164 bp double stranded DNA fragment that contained centrally the above preferred presumptive methylated site, and a 5′^32^P end label either at the top or the bottom strand so that nicking of both strands could be observed. The labelled DNA fragments were used to test for binding of Sco4631 using DNase I footprinting, and for detecting endonucleolytic activity. Figure 5A and 5B show a staggered cut in the position where four independent clones showed DNA cleavage (Figure 4C, third sequence). Overexposed gels showed two additional, faint bands corresponding to single strand nicks on the bottom strand only on the right side of the Dcm methylation site as it is shown in Figure 5B and 5C. All these bands were absent in the controls using an unmethylated form of the oligonucleotide or heat treated Sco4631.

There may be a trivial explanation for the lack of faint bands, indicating secondary cleavage sites in the upper strand: most the DNA molecules that were cleaved at one of the secondary sites in the upper strand will also be cleaved at the primary site. Because of the 5′ end-labeling, secondary cleavage cannot be detected.

DNase I footprinting revealed protection of 14 nucleotides centred around the Dcm methylation site C5mCTGG on the top strand (light blue nucleotides in Figure 5C), and weaker, asymmetric protection of the bottom strand containing the sequence C5mCAGG.

These results confirm that Sco4631 binds specifically to the Dcm methylated DNA and covers additional bases outside the C5mCWGG sequences.

### His-tagged Sco4631 protein cleaves *in vivo* S-modified DNA

*S. lividans* DNA is S-modified (phosphorothioated) at specific positions. Only about one in 6000 DNA backbone phosphates contain sulfur in a non-bridging position. S-modification of *S. lividans* DNA seems to be incomplete, and certain sequences are preferentially S-modified. The *Streptomyces* multicopy plasmid pHZ209 contains such a preferentially modified site which was chosen for our study.

**Figure 6:**
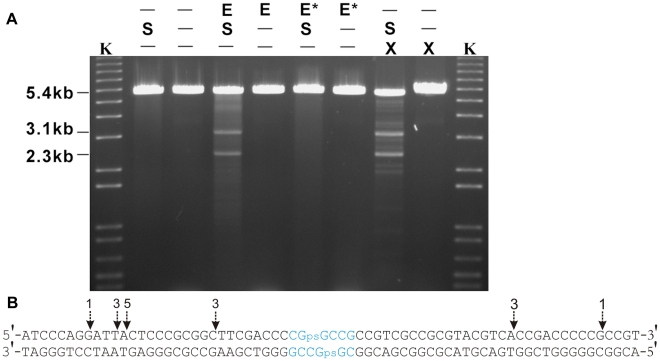
*In vitro* cleavage of S-modified (phosphorothioated) DNA by Tris-peracid or His_6_-Sco4631. A. Ethidium bromide-stained agarose gel showing EcoRV-linearized pHZ209 DNA (5.4 kb). K, kb ladder DNA size standard; S, S-modified DNA prepared from *S. lividans* 1326; E, His_6_-Sco4631; E*, inactive His_6_-Sco4631(H508A); X, Tris-peracid. Non-phosphorothioated DNA was prepared from *S. lividans* HXY6. Partial digestion producing strong 3.1 and 2.3 kb bands and additional fainter bands was observed in the samples containing S-modified DNA (S) and either His_6_-Sco4631 (E), or Tris-peracid. B. Summary of preferential cleavage sites on Dnd-phosphorothioated pHZ209. The two main bands (3.1 kb and 2.3 kb in Figure 5A) from the His_6_-Sco4631-digested S-modified DNA were cloned into pBluescript SK+, and 16 clones were end-sequenced from both sides using primers T3 and T7. Vertical arrows show where the cuts occurred, and the numbers indicate how many times each particular cut was observed. All 16 cuts occurred near the preferential Dnd-phosphorothioation modification sequence CGpsGCCG shown in blue.

S-modified pHZ209 was isolated from Dnd^+^ *S. lividans* 1326 and either cleaved oxidatively using Tris-peracid or using His-tagged Sco4631 protein and the same buffer that was used for the cleavage of Dcm methylated DNA. After digestion using EcoRV, which cuts pHZ209 once, three prominent bands and additional fainter bands were observed in both the Tris-peracid and the Sco4631-treated S-modified pHZ209 samples (Figure 6A). The largest fragment represents 5.4 kb linearised pHZ209 generated from plasmids that were not cleaved by Tris-peracid or Sco4631. The other two smaller bands were of the sizes expected if pHZ209 was cleaved at or near the preferential S-modification site. Controls with pHZ209 that was isolated from a *dnd^−^* host did not produce these two smaller bands.

The precise Sco4631 cleavage sites were determined by blunt end cloning and sequencing. Tris-peracid cleaves the DNA backbone precisely at the site of the sulfur producing a 1 bp 5′ overhanging staggered cut [19], [21]. The Sco4631-induced cuts were found on both sides between 16 and 28 nt away from the S-modification (dotted line arrows in Figure 6B). This was consistent with the expectation that Sco4631, like other Type IV REases, bound to the S-modification but cleaved some distance away from the cleavage site [26]–[28]. This endonucleolytic cleavage of DNA is, of course, consistent with our initial observations that Sco4631 and *dndA-E* are unable to coexist in the same host.

### Sco4631 cleaves DNA at multiple possible sites on either side of the S-modification

Oxidative cleavage of S-modified DNA breaks specifically the phosphorothioate bonds resulting in a 1 bp 5′ staggered cut. The above sequence data (Figure 6) suggested that Sco4631 cuts S-modified DNA rather imprecisely on either side near the sequence CGpsGCCG. The fact that cleavage occurred on both sides of the sequence suggests that Sco4631 may bind to both strands equally, i.e. the essential binding region may not extend beyond this palindromic sequence.

We could not exclude the possibility that the DNA propagated in *S. lividans* 1326 contained additional modifications that influenced the results. For this reason, we synthesized a 118 bp double-stranded oligonucleotide containing one phosphorothioate on each strand at the preferred sequence of pHZ209. Again, 5′^32^P label was used separately on both the top and bottom strand so that cuts in either strand could be observed. Oligonucleotides without S-modified bases were used as controls.

**Figure 7:**
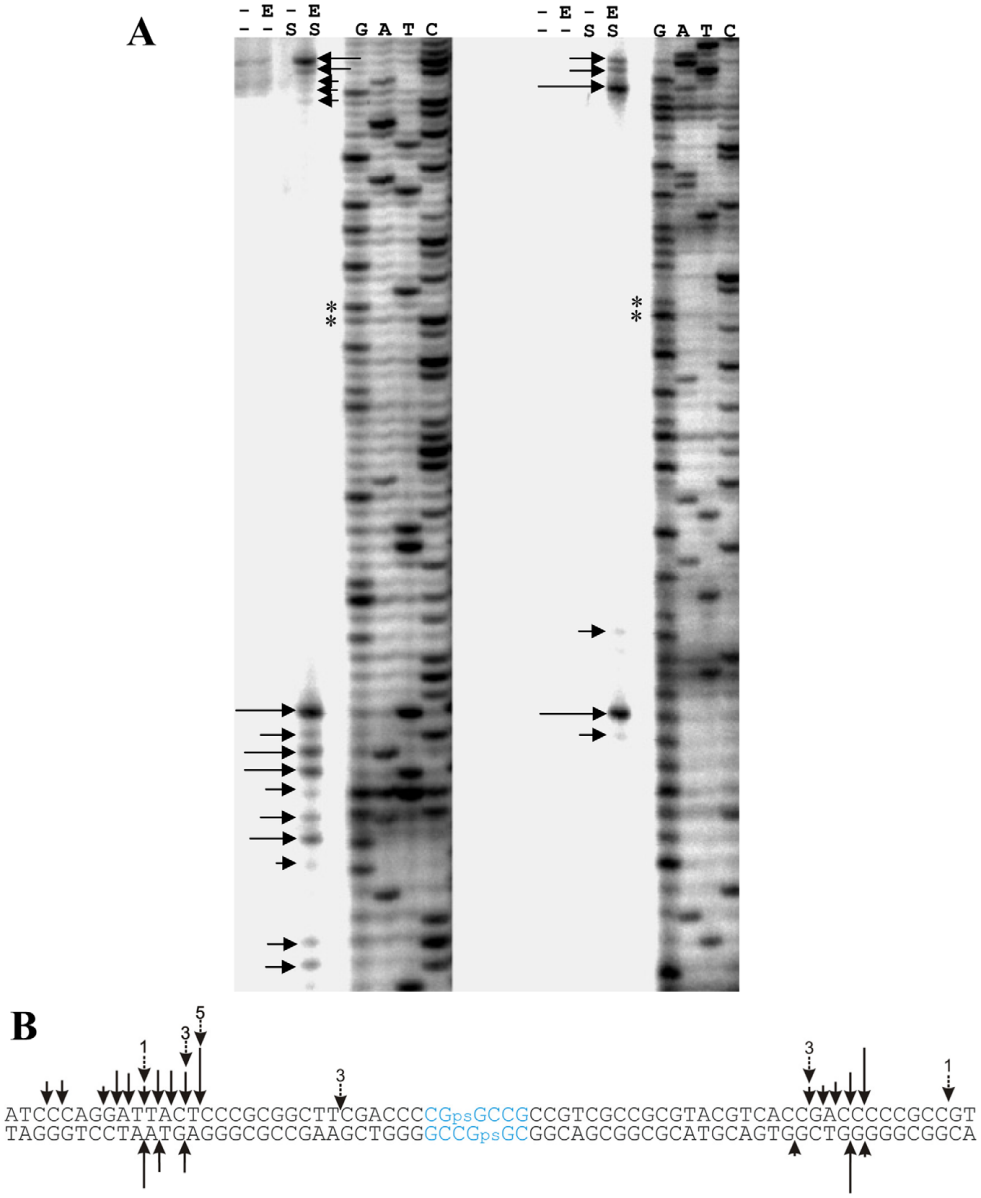
*In vitro* cleavage of a synthetic S-modified (phosphorothioated) double-stranded 118 bp oligonucleotide by purified His_6_-Sco4631. A. Autoradiographs of 5′ labeled top strand DNA (left) and 5′ labeled bottom strand DNA (right). G, A, T, C denote the sequencing ladders. Horizontal arrows indicate cleavage sites, and ** indicates the tandem G residues linked by a phosphorothioate bond. His_6_-Sco4631-mediated cleavage was observed exclusively in the samples of S-modified DNA (S) that also contained active His_6_-Sco4631 (E). Samples without DNA S modification or containing heat-inactivated His_6_-Sco4631 were not cleaved. - indicates the absence of enzyme or S-modification, respectively. B. *In vitro* cleavage of S-modified DNA by His_6_-Sco4631. Vertical arrows indicate bonds where cleavage of the 118 bp oligonucleotide was observed. The length of the solid arrows indicates the intensity of the bands in panel A and thus the frequency of cleavage. Dotted arrows indicate *in vitro* generated cleavage sites of pHZ209 (see Figure 5). The preferred S-modification sequence CGpsGCCG is shown in blue.

His-tagged Sco4631-specific cleavage was observed in both the top and the bottom strand, and on both sides of the S-modification (Figure 7). (Note that no cleavage occurred in the controls or at the site of S-modification which is marked by ** in Figure 7A and 7B.) Multiple cleavage sites were observed (solid arrows in Figure 7A and 7B), consistent with the data from the cloning and sequencing of individual cleavage sites (dotted arrows).

Surprisingly, no concentration dependent protection by Sco4631 of the S-modified DNA against DNase I was observed (Figure S6), possibly because the S-modified DNA was cleaved too efficiently. This result may not apply precisely to the majority of S-modified sites because this preferred site is a 6 bp palindrome rather than the more usual 4 bp core palindrome. Also, the S-modification in the synthetic oligonucleotides is racemic while the DndA-E proteins add the sulfur stereospecifically in the R_P_ configuration [18].

These results, however, confirmed that His_6_-tagged Sco4631 could cleave S-modified without interference from hypothetical DNA modifications that might have been present on naturally S-modified pHZ209.

## Discussion

### Activity of Sco4631

We demonstrated that the *S. coelicolor* protein Sco4631 is a Type IV REase that cleaves Dcm-methylated and also S-modified (phosphorothioated) DNA *in vitro* near the respective modification sites. DNA cleavage required a special reaction buffer containing Mn^2+^ or Co^2+^ at pH 9.0. These conditions reduce the sequence specificity of some Type II REases [24], [25]. Also the N-terminal His_6_ tag that was added to facilitate protein purification might have reduced or even altered the sequence specificity of the protein that was used for the *in vitro* studies. The fact that all the observed DNA cleavage events occurred very specifically near the respective modification sites suggests that neither the chosen buffer nor the His_6_ tag changed the enzyme specificity. Two other Type IV REases also have special buffer requirements for *in vitro* activity: GmrSD, encoded by an *E. coli* prophage, required Ca^2+^ and UTP for DNA cleavage [5], McrBC required GTP and Mg^2+^ for DNA binding [29]. Recently, Chan et al. reported that HNH nucleases generally have unusual buffer requirements [30]. Only MspJI from *Mycobacterium*, a remote homologue of *E. coli* Mrr, cleaved methylated DNA in a standard REase buffer [31]. (Please note that BseMII and BspLU11III have been reclassified as Type IIG REases [4], [32].The *in vivo* Dcm-methylated or S-modified DNAs might have contained unknown additional modifications that could have influenced the *in vitro* target selection. We excluded this possibility by repeating the cleavage experiments using long (164 and 118 bp) double-stranded synthetic oligonucleotides that had the same sequence and DNA modifications as the preferred cleavage sites identified on *in vivo* methylated or S-modified DNA. The results confirmed the initial data, indicating that there had been no unknown *in vivo* modifications influencing our results. In addition, using the end-labelled modified oligonucleotides provided better information about the relative frequencies of DNA cleavage in different positions (Figure 5, Figure 7).

We have thus shown that a few Dcm-methylated sites and one preferentially S-modified DNA sequence were cleaved by Sco4631. Gonzalez-Ceron et al. demonstrated, however, that also other sequence specific 5mC methylations lead to *in vivo* restriction in *Streptomyces* strains expressing Sco4631 [9]. We cannot exclude that Sco4631 can cleave DNA with methylated bases or S-modifications in many different possible target sequences. The observation that one of the six available Dcm sites in pOJ260 was cleaved four times while others were cleaved once or not at all could be due to statistical fluctuation, but it could also indicate that the uncut sites may have been undermethylated or that the DNA sequence around the methylation may influence the frequency of cleavage. It is also not possible to speculate about the importance of the surrounding DNA sequence for the enzymatic cleavage near S-modification sites because only a minority of the potential sites receive the sulfur, and some preferred sites are more strongly phosphorothioated than others [33]. Preferential S-modification alone, without differential cutting of sites depending on the surrounding DNA sequence, could have produced the major and minor bands in Figure 7A.

Type IV REases generally have low sequence specificity, and the same enzyme may cleave e.g. methylated and hydroxyl-methylated DNA [17], [27], [34]. A good illustration for this is MspJI that cleaved CmCWGG, CmCGG, AGmCT, GGmCC, GmCGC and mCCGG [31].

Gonzalez-Ceron et al. observed that a *S. lividans* strain expressing cloned *sco4631* restricted pSET152 DNA methylated by Dam or M. TaqI 400-fold and 20-fold, respectively, while Dcm methylated pSET152 was restricted only four fold [9]. It therefore seemed surprising at first that we could introduce pET28a::HisSco4631 into the Dam^+^Dcm^−^ strain BL21(DE3), but not into the Dam^+^Dcm^+^ strain DH10B. Tighter regulation of the *lac* promoter in the latter strain may have caused the difference rather than the methylation status.

The purified His_6_-Sco4631 was produced in *E. coli* that did not contain any other *Streptomyces* genes. This proved that Sco4631 acted without accessory proteins as are required for endonucleases activity by the McrBC family of Type IV REases [5], [29].

Sco4631 cleaved the Dcm-modified DNA preferentially to the left of the modification site on the sequence shown in Figure 5, indicating that the sequence flanking the 5mC modification may influence where cleavage occurs. The S-modified DNA was cleaved more evenly on both sides of the S-modification (Figure 7), but there was no evidence for simultaneous cleavage on both sites.

It was surprising that the distance between the DNA modification and the cleavage sites was different for Dcm and S-modified DNA. Maybe the enzyme remains locked to the Dcm site producing a near cut 12–16 bp away, but moves away from the S-modified site before cleaving less precisely c. 16–28 bp away.

The above speculation would be consistent with the observation that DNase I protection footprints, which were made under conditions that allowed DNA cleavage, were detectable on both strands of the Dcm-methylated oligonucleotides, but they were absent from the S-modified oligonucleotides (Figure S6). Note that the protection on each strand was asymmetric with respect to the methylated C.

The natural *S. lividans* DNA S-modification is chiral (P-S_R_-configuration, [18]) but the S-modification on the synthetic oligonucleotides was racemic and thus only one in four double-stranded oligonucleotides had the natural R-R conformation. It is unknown whether this may have affected protein binding or cleavage activity. The larger distance between the S-modification and the cleavage sites is, however, not an artefact of racemic S-modification because the larger distances were observed both with the naturally S-modified DNA and the synthetic S-modified oligonucleotide (see Figure 7B dotted arrows and solid arrows, respectively, for comparison).

Sco4631 had been identified by Gonzalez-Ceron et al. as a methyl-specific REase that has some limited (37% in a region of 91 aa) amino acid sequence similarity to the 5mC and 5hmC-specific EcoKMcrA [35], [36]. The two enzymes share a consensus HNH protein sequence motif that occurs in homing endonucleases and inteins, and in Group I and Group II introns [37], [38]. In REBASE [4] Sco4631 is listed as ScoA3McrA. It should be noted, however, that EcoKMcrA does not restrict Dcm methylated DNA [16].

### Biological functions of Sco4631

Because *in vitro* cleavage by Type IV REases has been elusive for many years, it was speculated that these enzymes may achieve restriction of modified DNA without DNA cleavage that could easily be reversed by re-ligation. Simple binding to the target DNA might prevent its replication or integration into the host genome [39]. By adding a third example of *in vitro* DNA cleavage by a Type IV REase we support the original idea that Type IV REases cleave DNA like all the other types of REases.

*S. coelicolor* restricts Dam, Dcm, Hsd and other methylated DNA very effectively using at least four different methyl-specific REases including Sco4631 [9], [40]. However, *S. coelicolor* only weakly restricts S-modified DNA from *S. lividans* [41]. Unexpectedly, the integrating vector pHZ1904 was severely restricted by *S. coelicolor* even though it did not have any known DNA modification because it had been propagated in the non-methylating *E. coli* host ET12567 that also did not support expression of the *dndA-E* DNA S-modification genes. Mutant derivatives of pHZ1904 were, however not restricted, excluding the possibility of an unknown sequence-specific (as opposed to modification-specific) restriction system acting in *S. coelicolor*.

It seemed therefore likely that expression of the *dndA-E* gene cluster in *S. coelicolor* resulted in S-modification of the host DNA. Sco4631 would then cleave near the modified sites resulting in cell death.

*S. coelicolor* mutants that tolerated S-modification by DndA-E represented about 50% of the rare pHZ1904 exconjugants. We speculated that one of the putative *S. coelicolor* endonucleases without counterpart in *S. lividans* might cleave S-modified DNA. Such a nuclease was indeed found to reside on the genomic island Gi11 [12]. Gi11, also known as SLP1 is a 17.1 kb mobile element that can excise from the *S. coelicolor* genome and transfer by conjugation to *S. lividans* where it usually forms autonomously replicating plasmids [14]. These plasmids in *S. lividans* always lack a part of the original Gi11 sequence including *sco4631* which would destroy the S-modified *S. lividans* DNA.

Occasionally, the entire SLP1 sequence integrates into the *S. lividans* genome [42]. It is not known whether these integrated elements feature a mutant *sco4631* or whether *dndA-E* is inactivated or deleted from these strains.

It seems that Sco4631 does not effectively restrict the entry of S-modified DNA into *S. coelicolor* [43], but it very effectively prevents the establishment of mobile elements that contain gene clusters like *dndA-E* that cause S-modification of its DNA. Such suicidal processes are frequently used by plasmids containing stable *kil* (kill) and unstable *kor* (kill override) genes resulting in cell death as a result of plasmid loss [44]. Also, restriction-modification (R-M) systems which are often on mobile elements [45] are stabilized in a bacterial population because the R-activity persists longer than the M-activity after the genes have been lost from a cell [46]. Fukuda et al. proposed that an important function of Type IV REases in general is the sacrificial killing of cells that have newly acquired a DNA modification system [39].

It was expected that strains containing a *dnd* gene cluster would not contain a Sco4631 homologue. This was true for 40 out of 41 fully sequenced strains containing a *dnd* gene cluster. *Pseudomonas fluorescens* Pf0-1, however, contains both a complete *dnd* gene cluster and S-modified DNA, and it also contains two HNH proteins that are, however, very different from Sco4631 (Figure S7A, S7B).

It is not understood why bacteria need such defences against DNA modification systems. Dam methylation reduces the mutation rate by directing the *mutHLS* pathway to correct errors in the newly synthesized DNA strand, and DNA adenine methylation also has known regulatory roles in bacteria [47]–[49], but the functions of Dcm methylation and phosphorothioation remain unknown.

### Importance of Sco4631 and DndA-E for the genomic islands Gi11 of *S. coelicolor* and SLG of *S. lividans*

SLP1 in *S. coelicolor* and SLG in *S. lividans* are segregationally very stable genomic islands [13], [50]. Spontaneous excision of each element in the absence of selective pressure could, however, be observed using sensitive PCR analysis (Figure S3B) [13].

Our experiments demonstrate that both SLP1 and SLG can be cleanly excised from the respective genomes by natural processes.

The sequence-specific S-modification of DNA prevents DNA cleavage by some Type II REases whose specificity overlaps the S-modification site (Liang J, personal communication), and *Salmonella enterica* serovar Cerro 87 which has S-modified DNA, also contains a restriction system that cleaves foreign DNA that lacks the cognate S-modification [51]. The fact that *S. coelicolor* has in Sco4631 an effective defence against invasion by these genes indicates that DNA S-modification may have important biological functions that remain to be discovered.

## Materials and Methods

Strains, plasmids and primers used are listed in Table S1 and Table S2, respectively. *E. coli* and *Streptomyces* strains were grown in LB [52] and SFM medium [11], respectively, supplemented with 100 µg/ml ampicilin, 30 µg/ml apramycin, or 12 µg/ml thiostrepton as required. Transformation of *E. coli* DH10B with *Streptomyces* plasmids was performed using the machine EasyjecT Plus electroporator (EQUIBIO). Conjugative transfer of DNA from *E. coli* ET12567/pUZ8002 to *Streptomyces* was performed as described in [11], except that always approximately 10^10^ recipient spores were used, and tenfold dilutions of donor cells (10^9^ to 10^5^ CFU) were used per 9 cm agar plate. The exconjugants were counted from appropriate dilutions after incubation at 30°C for 3 days on SFM plates. The number of *E. coli* and *Streptomyces* CFUs in each experiment was determined by making dilutions and plating LB agar and MS plates, respectively. The conjugation frequencies were expressed as the number of exconjugants per 10^9^ CFU of donors. The calcium chloride transformation frequency of *E. coli* was determined using 10 ng of CCC plasmid DNA. Each experiment was repeated three times and the final frequency was the mean value. Investigation of Dnd phenotype (degradation of S-modified DNA by Tris-peracid) was carried out as described in [21].

### Inactivation and heterologous expression of *sco4631*

An internal region of *sco4631* was amplified by PCR using primers S31DF and S31DR, gel purified and inserted into the EcoRV site of pBluescript prior to transformation into pSET151 to give pJTU1653, which was then transferred from ET12567/pUZ8002 into *S. coelicolor* M145 by two-parental mating [11]. A 2176 bp fragment containing intact *sco4631* was amplified using KOD Plus (TOYOBO) and primers S31HEF (XbaI linker) and S31HER (XbaI linker). The resulting fragment was cut by XbaI and inserted into pBluescript, and then the XbaI insertion was purified from this intermediate and cloned into the XbaI site of pPM927 to give pJTU1654. pJTU1654 and pPM927 were transformed individually into ET12567/pUZ8002 for conjugation into *S. lividans* strains 1326 and HXY16.

### Overexpression and purification of His-tagged Sco4631 and its mutant

*sco4631* was amplified by PCR using KOD Plus and primers S31OEF and S31OER, inserted into pBluescript, excised as an NdeI–EcoRI fragment and ligated between cognate sites of pET28a, generating pJTU1655. Site directed mutagenesis of pJTU1655 was carried out by using KOD-Plus-Mutagenesis Kit (TOYOBO) with primers H508A-F and H508A-R, resulting in pSco4631H508A. The expression constructs were then transformed into BL21(DE3)/pLysE respectively. 10 ml of the overnight culture of BL21(DE3)/pLysE pJTU1655 or BL21(DE3)/pLysE pSco4631H508A was inoculated into 1 L LB medium supplied with 50 µg/ml kanamycin and 34 µg/ml chloramphenicol. Then the culture was incubated at 37°C to OD_600_  = 0.4, cooled to room temperature and 0.4 mM IPTG was added, followed by incubation for another 5 hours at 30°C. Then the cells were harvested and resuspended in 20 ml binding buffer (20 mM Tris-Cl, 20 mM imidazole and 150 mM NaCl pH 8.0) and lysed by sonication in an ice bath. After centrifugation (16000 g for 30 min at 4°C), the supernatant was applied to a HisTrap HP column (GE Healthcare) and purified using an ÄKTA FPLC (GE Healthcare) by eluting with imidazole linear gradient 20–500 mM. The product was desalted by a HiTrap Desalting column (GE healthcare) and stored in Tris-Cl buffer (50 mM, pH 8.0) with 50% glycerol at −20°C. Purified Sco4631 was visualized by Coomassie-stained 12% SDS-PAGE analysis and protein concentration determined using a Bradford Protein Assay Kit (Bio-Rad). The protein purity was determined by Quantity One (Bio-Rad) from the gel, and the purities of Sco4631 and Sco4631(H508A) are about 96.5% and 97.9%, respectively.

### Optimization of in vitro conditions for cleavage activity of Sco4631

Total *S. lividans* 1326 DNA prepared by the Kirby mix procedure was used as a substrate for the Sco4631 cleavage activity assay. Purified Sco4631 protein (500 ng) and 100 ng DNA substrate were incubated at 30°C for 5 min in a total reaction volume of 20 µl. The reaction buffer contained 20 mM Tris-Cl (pH 8.0), 50 mM NaCl and each of seven divalent ions, Ca^2+^, Co^2+^, Cu^2+^, Mg^2+^, Mn^2+^, Ni^2+^ or Zn^2+^ at a concentration of 1 nM to 10 mM. The reactions were stopped by adding loading buffer (Takara) containing 1% SDS, 50% glycerol and 0.05% bromophenol blue. The DNA after the reaction was examined by 0.75% agarose gel electrophoresis. Mn^2+^ or Co^2+^ ions at concentrations between 100 µM and 10 mM gave maximal DNA cleavage. The optimal pH was determined in a similar manner using equivalent buffers of pH 5 to pH 10, supplemented with 1 mM Mn^2+^; pH 9 was optimal for Sco4631-mediated DNA cleavage.

### End sequencing of fragments generated by Sco4631 cleavage of Dcm-methylated or Dnd-phosphorothioated DNA

The plasmids pOJ260 and pHZ209 were isolated from DH10B and *S. lividans* 1326, respectively, using the QIAGEN Plasmid Midi Kit (Qiagen). One microgram pOJ260 DNA was incubated with Sco4631 under the optimized conditions described above. Linearised pOJ260 was gel purified and blunted using Klenow Fragment (Fermentas) and dNTPs at 37°C for 10 min in a total volume of 20 µl (1×reaction buffer, 0.05 mM dNTP mix, 5 Unit Klenow Fragment), followed by heating at 75°C for 10 min to stop the reaction. The blunted mix was ligated to a 1311 bp amplicon harboring a *bla* cassette and the ligation mix was transformed into *E. coli* DH10B. Plasmid DNA was then prepared from randomly selected ampicillin-resistant transformants for insert end-sequencing using the primers seqF and seqR. Similarly, pHZ209 was linearized using EcoRV and then incubated with Sco4631. The resulting DNA fragments were gel purified, blunted using Klenow fragment, and ligated into the EcoRV site of pBluescript for transformation into *E. coli* DH10B. Plasmid DNA was then prepared from randomly selected transformants for insert end-sequencing using the primers T3 and T7.

### Generation of 5′ end-labeled methylated or phosphorothioated DNA substrates for cleavage and DNase I footprinting analyses

A 164 bp PCR amplicon was generated using pOJ260 as a template, and the long primers MF and MR that contained a m5C residue on each strand within the C^m5^C(A/T)GG Dcm-modification motif. An unmodified matching amplicon was also generated using primers UMF and UMR. Individual strands of the modified and unmodified amplicons were 5′ end-labeled by pre-tagging the appropriate primer with γ-^32^P using the following protocol. 10 pmol of primer was incubated with [γ-^32^P]-ATP (Beijing Furui Co. Ltd) using 10U of T4 polynucleotide kinase (Promega) at 37°C for 30 min, followed by 90°C for 2 min to inactivate the T4 polynucleotide kinase. PCR reactions were performed in a total volume of 50 µl containing 2 ng template DNA, 10 pmol of each primer, 1× PCR buffer, 5% DMSO, and 5U Taq polymerase (Genescript). PCR products were purified using the QIAquick PCR Purification Kit (Qiangen). An identical strategy was used to synthesize and strand-specifically label the phosphorothioate-modified and unmodified pHZ209-derived 118 bp amplicons. The phosphorothioate modification was introduced between the tandem guanine residues in the Dnd-motif (GpsGCC) by prior chemical synthesis of the long PCR primers SF and SR. The matching unmodified amplicon was generated using the primers USF and USR.

### Cleavage sites analysis and DNase I footprinting assay

The DNA substrates used in these analyses were as described above. For cleavage sites analysis, the reaction mixture containing 100 cps ^32^P-labeled DNA substrate (10 nM), 20 mM Tris-Cl (pH 9.0), 50 mM NaCl, 1 mM MnCl_2_, 5% glycerol and 500 ng Sco4631 in a total volume of 20 µl was incubated at 30°C for 5 min. For the DNase I footprinting assay, 500 cps ^32^P-labeled DNA substrate (50 nM) was added to 0–24 µg (18 µM) of Sco4631 under the same buffer conditions as above, and the reaction mix was then incubated on ice for 5 min prior to adding 2.5 µl DNase I buffer and 0.3 U of DNase I (Promega), and further incubation at 37°C for 1 min. The reaction was stopped by adding 100 µl stop solution (3 M ammonium acetate, 0.25 M EDTA, 1 mg/ml glycogen), and 50 µl phenol-chloroform. Samples were then denatured at 95°C for 2 min and loaded on an 8% polyacrylamide–urea gel. The DNA sequence ladder was generated using an *fmol* DNA Cycle Sequencing kit (Promega). After electrophoresis, the gels were dried and exposed to a Kodak X-ray film.

### Accession number

The nucleotide sequence of pOJ260 has been deposited in GenBank under Accession number GU270843.

## Supporting Information

Figure S1 Half of the *S. coelicolor* exconjugants contain mutated *dnd* gene clusters. A. Ethidium bromide-stained agarose gel showing total genomic DNA of *S. coelicolor* exconjugants run under conditions that favor the Tris-peracid-mediated cleavage of DNA phosphorothioate bonds. The exconjugants in lanes 1, 6, 7, 8, 10 and 11 had stable DNA and thus exhibited the Dnd^−^ phenotype associated with DNA without S-modification. The DNA in lanes 2, 3, 4, 5 and 9 was visibly degraded (characteristic DNA smear) and thus exhibited the Dnd^+^ phenotype associated with S-modified (phosphorothioated) DNA. B. Organisation of the *dndA-E* gene cluster and the structure of mutant gene clusters from three randomly selected Dnd^−^ (without S) exconjugants. The bold C denotes the single-nucleotide insertion in pHZ1904*. Another isolate contained a *Tn*10 insertion (acquired in *E. coli*) in *dndC*, and the third isolate had suffered a deletion removing *dndB-E*.
(0.71 MB TIF)

Figure S2 Construction of the *sco4631* mutant strain. An 1438 bp internal fragment of *sco4631* was generated by PCR amplification using primers S31DF and S31DR (red arrows). This fragment was inserted into the suicide vector pSET151. Introduction of this construct into *S. coelicolor* M145 and thiostrepton selection resulted in single crossover integration into *sco4631*. The resulting strain LG4 lacked a functional copy of *sco4631*. *tsr*, thiostrepton resistance gene for selection in *Streptomyces*.
(0.10 MB TIF)

Figure S3 Confirmation that the genomic islands Gi11 ( = SLP1, encoding *sco4631* near *attR*) and SLG (containing *dndA-E*) were precisely deleted from the *S. coelicolor* and *S. lividans* genomes, respectively. A. Gi11 resides in a tRNA*^Tyr^* sequence (yellow box) in *S. coelicolor*. The insertion regenerated tRNA*^Tyr^* at attL and created a 112 bp direct repeat at *attR* (black triangles). The primers UF and DR from outside the direct repeat were used to detect samples from which the Gi11 sequence had been deleted. The ethidium bromide-stained agarose gel shows the characteristic 1497bp PCR fragment obtained with *S. lividans* 1326 DNA (lane 2) and ten *S. coelicolor* strains that expressed the cloned *dndA-E* gene cluster cloned on pHZ1904 (lanes 3–12). Lane 1 shows that excision of Gi11 was not detectable in wild-type *S. coelicolor* M145. B. The genomic island SLG of *S. lividans* is also flanked by 15 bp direct repeats (green triangles). The primers LP1F and Rp1R from outside the direct repeats were used to detect excision of SLG. The ethidium bromide-stained agarose gel to the right shows that the characteristic 725 bp band was obtained using *S. coelicolor* M145 DNA (land 1) and DNA from ten *S. lividans* derivatives expressing the *S. coelicolor* gene *sco4631* (lanes 3–12). No such band was observed with DNA from wild-type *S. lividans* 1326 (lane 2).
(0.61 MB TIF)

Figure S4 Transfer of *sco4631* from *E. coli* to *S. lividans* strains. HXY16 is the *S. lividans* derivative which lacks the *dnd* gene cluster; pPM927-sco4631 (pJTU1654) is pPM927 harboring *sco4631* with its native promoter. *E. coli* strain used for conjugation is ET12567::pUZ8002.
(0.15 MB TIF)

Figure S5 Heterologous expression of His6-tagged Sco4631 and its mutant in *E. coli*. Sco, protein eluted from the Ni affinity column as a 64.2 kd polypeptide. M, size markers.
(0.67 MB TIF)

Figure S6 DNase I protection assay of a synthetic S-modified (phosphorothioated) double-stranded 118 bp oligonucleotide by purified His6-Sco4631. Autoradiographs of 5′ labeled top strand DNA (left) and 5′ labeled bottom strand DNA (right). G, A, T, C denote the sequencing ladders. ** indicates the tandem G residues linked by a phosphorothioate bond. Lanes 1–4 are controls containing unphosphorothioated DNA and increasing amounts of active His6-Sco4631. Lanes 5–8 contained phosphorothioated DNA and increasing amounts of the active His6-Sco4631. Lanes 1 and 5 contained no enzyme, and lanes 2–4 and lanes 6–8 contained 1.1, 4.5 and 18 µM enzyme, respectively.
(3.20 MB TIF)

Figure S7 Phylogenetic analysis of Sco4631. A. Unrooted phylogenetic tree showing the extreme diversity of Sco4631 and its closest homologues. The 560 aa sequence of Sco4631 was used as a template to search REbase and the non-redundant protein database using BlastP for Type IV REases. The 59 top scoring sequences (E<10^−5^) were aligned using ClustalW. The scale represents an evolutionary distance of 0.1. Red, ScoA3McrA = Sco4631 is most similar to the 481 aa conserved hypothetical protein SSEG03462 of *Streptomyces sviceus* ATCC 29083 (389/466 = 83% identity) a producer of many oxidative antibiotic tailoring enzymes. The next closest homologue is the putative HNH endonuclease SkeORF2910P from the Actinomycete *Sanguibacter keddieii* DSM 10542 which was isolated from bovine blood. Purple, EcoKMcrA from the e14 prophage in *E. coli* K-12, and from three other *E. coli* strains containing the e14 prophage. EcoKMcrA has an evolutionary distance >0.8 from ScoA3McrA, and only 34/91 aa identity in the HNH region. Green, two putative HNH endonucleases from *Pseudomonas fluorescens* Pf0-1 which contains a complete *dnd* gene cluster and S-modified (phosphorothioated) DNA. This strain was thus not expected to contain a protein that cleaves S-modified DNA. The evolutionary distance of these proteins is about 1, and the identity is only about 40% in a 65 aa region containing the HNH conserved sequence motif. One of the proteins (McrA2P) may be inactive because it lacks two highly conserved amino acids (GE) in the centre of the HNH motif (Hx13Nx8H; see Figure McrA HNH alignments). Note, 22 of the 59 Esa proteins are from environmental DNA samples. B. Mutiple alignment of ScoA3McrA and the 58 most similar proteins. The dots above the sequence mark H, N and H of the conserved Hx13Nx8H (HNH) motif. Arrows to the left mark sequences from *Pseudomonas fluorescens* Pf0-1 which contains S-modified DNA. The deletion of two amino acid residues in PflCMcrA2P are marked with blue rectangle.
(2.00 MB TIF)

Table S1 Strains and plasmids used in this study.
(0.06 MB DOC)

Table S2 Primers used in this study.
(0.05 MB DOC)

## References

[1] Robertson KD (2005). DNA Methylation and human disease.. Nat Rev Genet.

[2] Bickle TA, Kruger DH (1993). Biology of DNA restriction.. Microbiol Rev.

[3] Dryden DT, Murray NE, Rao DN (2001). Nucleoside triphosphate-dependent restriction enzymes.. Nucleic Acids Res.

[4] Roberts RJ, Vincze T, Posfai J, Macelis D (2010). REBASE–a database for DNA restriction and modification: enzymes, genes and genomes.. Nucleic Acids Res.

[5] Bair CL, Black LW (2007). A type IV modification dependent restriction nuclease that targets glucosylated hydroxymethyl cytosine modified DNAs.. J Mol Biol.

[6] Zhou X, He X, Liang J, Li A, Xu T (2005). A novel DNA modification by sulphur.. Mol Microbiol.

[7] Zhou X, Deng Z, Firmin JL, Hopwood DA, Kieser T (1988). Site-specific degradation of *Streptomyces lividans* DNA during electrophoresis in buffers contaminated with ferrous iron.. Nucleic Acids Res.

[8] Bentley SD, Chater KF, Cerdeno-Tarraga AM, Challis GL, Thomson NR (2002). Complete genome sequence of the model actinomycete *Streptomyces coelicolor* A3(2).. Nature.

[9] Gonzalez-Ceron G, Miranda-Olivares OJ, Servin-Gonzalez L (2009). Characterization of the methyl-specific restriction system of *Streptomyces coelicolor* A3(2) and of the role played by laterally acquired nucleases.. FEMS Microbiol Lett.

[10] Flett F, Mersinias V, Smith CP (1997). High efficiency intergeneric conjugal transfer of plasmid DNA from *Escherichia coli* to methyl DNA-restricting streptomycetes.. FEMS Microbiol Lett.

[11] Kieser T, Bibb MJ, Chater KF, Butter MJ, Hopwood DA (2000). Practical *Streptomyces* genetics. a laboratory manual..

[12] Jayapal KP, Lian W, Glod F, Sherman DH, Hu WS (2007). Comparative genomic hybridizations reveal absence of large *Streptomyces coelicolor* genomic islands in *Streptomyces lividans*.. BMC Genomics.

[13] He X, Ou HY, Yu Q, Zhou X, Wu J (2007). Analysis of a genomic island housing genes for DNA S-modification system in *Streptomyces lividans* 66 and its counterparts in other distantly related bacteria.. Mol Microbiol.

[14] Bibb MJ, Ward JM, Kieser T, Cohen SN, Hopwood DA (1981). Excision of chromosomal DNA sequences from *Streptomyces coelicolor* forms a novel family of plasmids detectable in *Streptomyces lividans*.. Mol Gen Genet.

[15] Raleigh EA, Wilson G (1986). *Escherichia coli* K-12 restricts DNA containing 5-methylcytosine.. Proc Natl Acad Sci U S A.

[16] Mulligan EA, Dunn JJ (2008). Cloning, purification and initial characterization of *E. coli* McrA, a putative 5-methylcytosine-specific nuclease.. Protein Expr Purif.

[17] Mulligan EA, Hatchwell E, McCorkle SR, Dunn JJ (2010). Differential binding of *Escherichia coli* McrA protein to DNA sequences that contain the dinucleotide m5CpG.. Nucleic Acids Res.

[18] Wang L, Chen S, Xu T, Taghizadeh K, Wishnok JS (2007). Phosphorothioation of DNA in bacteria by *dnd* genes.. Nat Chem Biol.

[19] Ray T, Mills A, Dyson P (1995). Tris-dependent oxidative DNA strand scission during electrophoresis.. Electrophoresis.

[20] Ou HY, He X, Shao Y, Tai C, Rajakumar K (2009). *dnd*DB: a database focused on phosphorothioation of the DNA backbone.. PLoS ONE.

[21] Liang J, Wang Z, He X, Li J, Zhou X (2007). DNA modification by sulfur: analysis of the sequence recognition specificity surrounding the modification sites.. Nucleic Acids Res.

[22] Sun Y, He X, Liang J, Zhou X, Deng Z (2009). Analysis of functions in plasmid pHZ1358 influencing its genetic and structural stability in *Streptomyces lividans* 1326.. Appl Microbiol Biotechnol.

[23] Evans M, Kaczmarek FS, Stutzman-Engwall K, Dyson P (1994). Characterization of a *Streptomyces-lividans*-type site-specific DNA modification system in the avermectin-producer *Streptomyces avermitilis* permits investigation of two novel giant linear plasmids, pSA1 and pSA2.. Microbiology.

[24] Hsu M, Berg P (1978). Altering the specificity of restriction endonuclease: effect of replacing Mg^2+^ with Mn^2+^.. Biochemistry.

[25] Thielking V, Selent U, Kohler E, Landgraf A, Wolfes H (1992). Mg^2+^ confers DNA binding specificity to the EcoRV restriction endonuclease.. Biochemistry.

[26] Janulaitis A, Petrusyte M, Maneliene Z, Klimasauskas S, Butkus V (1992). Purification and properties of the Eco57I restriction endonuclease and methylase–prototypes of a new class (type IV).. Nucleic Acids Res.

[27] Stewart FJ, Raleigh EA (1998). Dependence of McrBC cleavage on distance between recognition elements.. Biol Chem.

[28] Jurenaite-Urbanaviciene S, Kazlauskiene R, Urbelyte V, Maneliene Z, Petrusyte M (2001). Characterization of BseMII, a new type IV restriction-modification system, which recognizes the pentanucleotide sequence 5′-CTCAG(N)(10/8).. Nucleic Acids Res.

[29] Stewart FJ, Panne D, Bickle TA, Raleigh EA (2000). Methyl-specific DNA binding by McrBC, a modification-dependent restriction enzyme.. J Mol Biol.

[30] Chan SH, Opitz L, Higgins L, O'Loane D, Xu SY (2010). Cofactor requirement of HpyAV restriction endonuclease.. PLoS ONE.

[31] Zheng Y, Cohen-Karni D, Xu D, Chin HG, Wilson G (2010). A unique family of Mrr-like modification-dependent restriction endonucleases.. Nucleic Acids Res.

[32] Roberts RJ, Belfort M, Bestor T, Bhagwat AS, Bickle TA (2003). A nomenclature for restriction enzymes, DNA methyltransferases, homing endonucleases and their genes.. Nucleic Acids Res.

[33] Dyson P, Evans M (1998). Novel post-replicative DNA modification in *Streptomyces*: analysis of the preferred modification site of plasmid pIJ101.. Nucleic Acids Res.

[34] Sutherland E, Coe L, Raleigh EA (1992). McrBC: a multisubunit GTP-dependent restriction endonuclease.. J Mol Biol.

[35] Raleigh EA, Trimarchi R, Revel H (1989). Genetic and physical mapping of the *mcrA* (*rglA*) and *mcrB* (*rglB*) loci of *Escherichia coli* K-12.. Genetics.

[36] Raleigh EA, Benner J, Bloom F, Braymer HD, DeCruz E (1991). Nomenclature relating to restriction of modified DNA in *Escherichia coli*.. J Bacteriol.

[37] Gorbalenya AE (1994). Self-splicing group I and group II introns encode homologous (putative) DNA endonucleases of a new family.. Protein Sci.

[38] Dalgaard JZ, Klar AJ, Moser MJ, Holley WR, Chatterjee A (1997). Statistical modeling and analysis of the LAGLIDADG family of site-specific endonucleases and identification of an intein that encodes a site-specific endonuclease of the HNH family.. Nucleic Acids Res.

[39] Fukuda E, Kaminska KH, Bujnicki JM, Kobayashi I (2008). Cell death upon epigenetic genome methylation: a novel function of methyl-specific deoxyribonucleases.. Genome Biol.

[40] MacNeil DJ (1988). Characterization of a unique methyl-specific restriction system in *Streptomyces avermitilis*.. J Bacteriol.

[41] Thompson CJ, Ward JM, Hopwood DA (1982). Cloning of antibiotic resistance and nutritional genes in streptomycetes.. J Bacteriol.

[42] Omer CA, Cohen SN (1984). Plasmid formation in *Streptomyces*: excision and integration of the SLP1 replicon at a specific chromosomal site.. Mol Gen Genet.

[43] Thompson CJ, Kieser T, Ward JM, Hopwood DA (1982). Physical analysis of antibiotic-resistance genes from *Streptomyces* and their use in vector construction.. Gene.

[44] Bahl MI, Hansen LH, Sorensen SJ (2009). Persistence mechanisms of conjugative plasmids.. Methods Mol Biol.

[45] Furuta Y, Abe K, Kobayashi I (2010). Genome comparison and context analysis reveals putative mobile forms of restriction-modification systems and related rearrangements.. Nucleic Acids Res.

[46] Asakura Y, Kobayashi I (2009). From damaged genome to cell surface: transcriptome changes during bacterial cell death triggered by loss of a restriction-modification gene complex.. Nucleic Acids Res.

[47] Collier J (2009). Epigenetic regulation of the bacterial cell cycle.. Curr Opin Microbiol.

[48] Marinus MG, Casadesus J (2009). Roles of DNA adenine methylation in host-pathogen interactions: mismatch repair, transcriptional regulation, and more.. FEMS Microbiol Rev.

[49] Modrich P (1987). DNA mismatch correction.. Annu Rev Biochem.

[50] Lee SC, Omer CA, Brasch MA, Cohen SN (1988). Analysis of recombination occurring at SLP1 *att* sites.. J Bacteriol.

[51] Xu T, Yao F, Zhou X, Deng Z, You D (2010). A novel host-specific restriction system associated with DNA backbone S-modification in *Salmonella*.. Nucleic Acids Res.

[52] Sambrook J, Fritsch EF, Maniatis T (1989). Molecular cloning: a laboratory manual, 2nd ed..

