# Supplementary figures and images for: Cleavage of Phosphorothioated DNA and Methylated DNA by the Type IV Restriction Endonuclease ScoMcrA
Source: PLoS Genet. 2010 Dec 23;6(12):e1001253. doi: 10.1371/journal.pgen.1001253 (PMC3009677; doi:10.1371/journal.pgen.1001253)

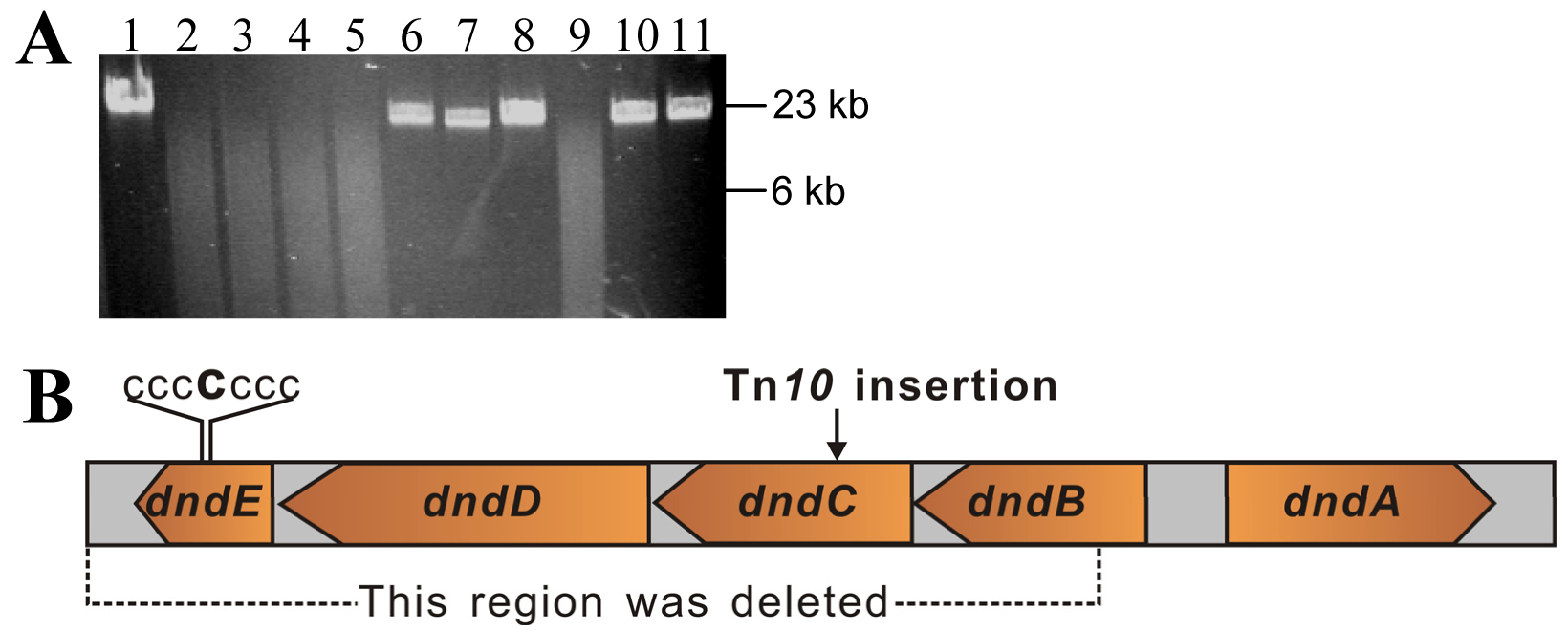

Supplement: Figure S1 — Half of the S. coelicolor exconjugants contain mutated dnd gene clusters. A. Ethidium bromide-stained agarose gel showing total genomic DNA of S. coelicolor exconjugants run under conditions that favor the Tris-peracid-mediated cleavage of DNA phosphorothioate bonds. The exconjugants in lanes 1, 6, 7, 8, 10 and 11 had stable DNA and thus exhibited the Dnd− phenotype associated with DNA without S-modification. The DNA in lanes 2, 3, 4, 5 and 9 was visibly degraded (characteristic DNA smear) and thus exhibited the Dnd+ phenotype associated with S-modified (phosphorothioated) DNA. B. Organisation of the dndA-E gene cluster and the structure of mutant gene clusters from three randomly selected Dnd− (without S) exconjugants. The bold C denotes the single-nucleotide insertion in pHZ1904*. Another isolate contained a Tn10 insertion (acquired in E. coli) in dndC, and the third isolate had suffered a deletion removing dndB-E. (0.71 MB TIF) [file pgen.1001253.s001.tif]

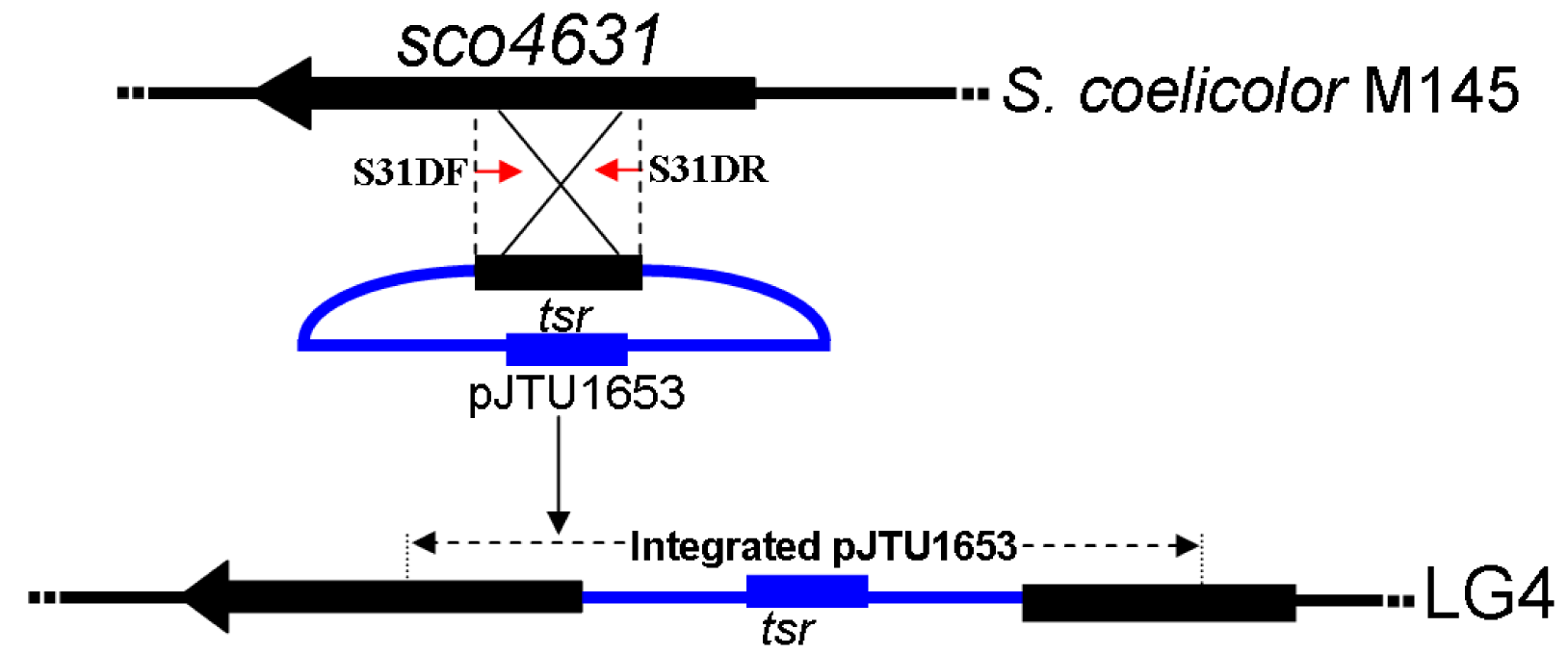

Supplement: Figure S2 — Construction of the sco4631 mutant strain. An 1438 bp internal fragment of sco4631 was generated by PCR amplification using primers S31DF and S31DR (red arrows). This fragment was inserted into the suicide vector pSET151. Introduction of this construct into S. coelicolor M145 and thiostrepton selection resulted in single crossover integration into sco4631. The resulting strain LG4 lacked a functional copy of sco4631. tsr, thiostrepton resistance gene for selection in Streptomyces. (0.10 MB TIF) [file pgen.1001253.s002.tif]

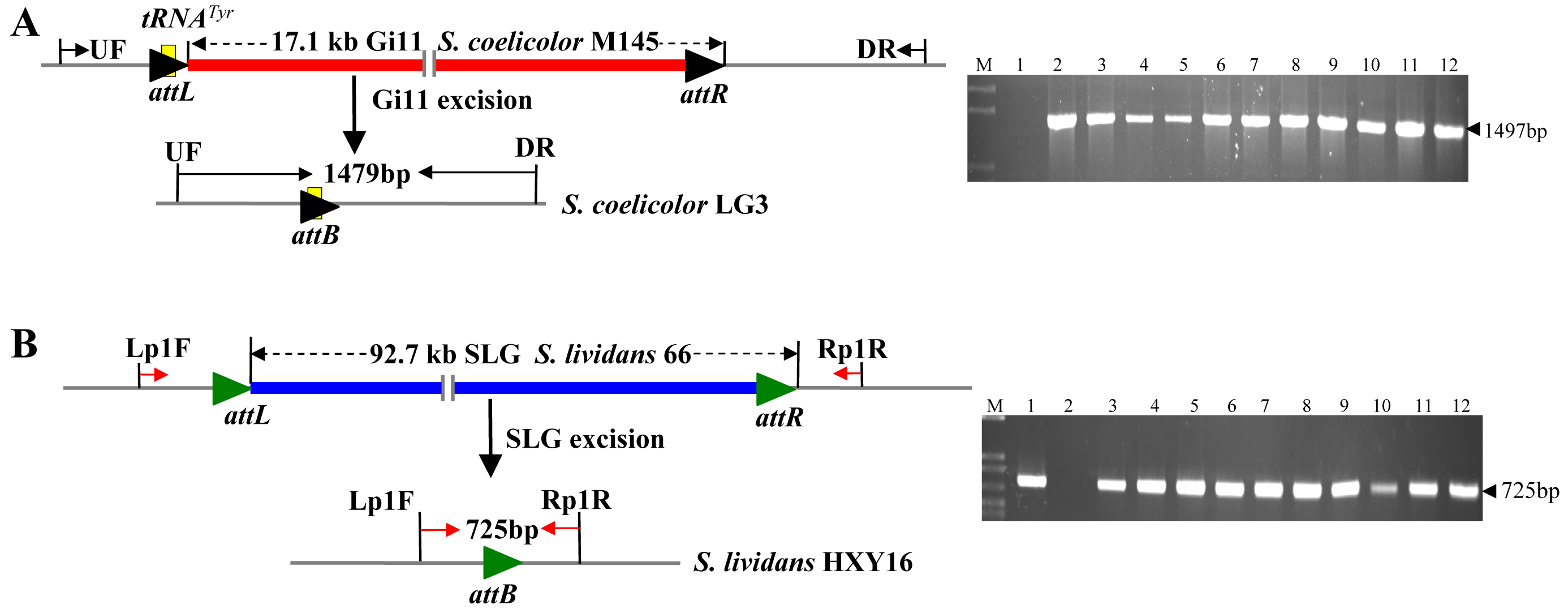

Supplement: Figure S3 — Confirmation that the genomic islands Gi11 ( = SLP1, encoding sco4631 near attR) and SLG (containing dndA-E) were precisely deleted from the S. coelicolor and S. lividans genomes, respectively. A. Gi11 resides in a tRNATyr sequence (yellow box) in S. coelicolor. The insertion regenerated tRNATyr at attL and created a 112 bp direct repeat at attR (black triangles). The primers UF and DR from outside the direct repeat were used to detect samples from which the Gi11 sequence had been deleted. The ethidium bromide-stained agarose gel shows the characteristic 1497bp PCR fragment obtained with S. lividans 1326 DNA (lane 2) and ten S. coelicolor strains that expressed the cloned dndA-E gene cluster cloned on pHZ1904 (lanes 3–12). Lane 1 shows that excision of Gi11 was not detectable in wild-type S. coelicolor M145. B. The genomic island SLG of S. lividans is also flanked by 15 bp direct repeats (green triangles). The primers LP1F and Rp1R from outside the direct repeats were used to detect excision of SLG. The ethidium bromide-stained agarose gel to the right shows that the characteristic 725 bp band was obtained using S. coelicolor M145 DNA (land 1) and DNA from ten S. lividans derivatives expressing the S. coelicolor gene sco4631 (lanes 3–12). No such band was observed with DNA from wild-type S. lividans 1326 (lane 2). (0.61 MB TIF) [file pgen.1001253.s003.tif]

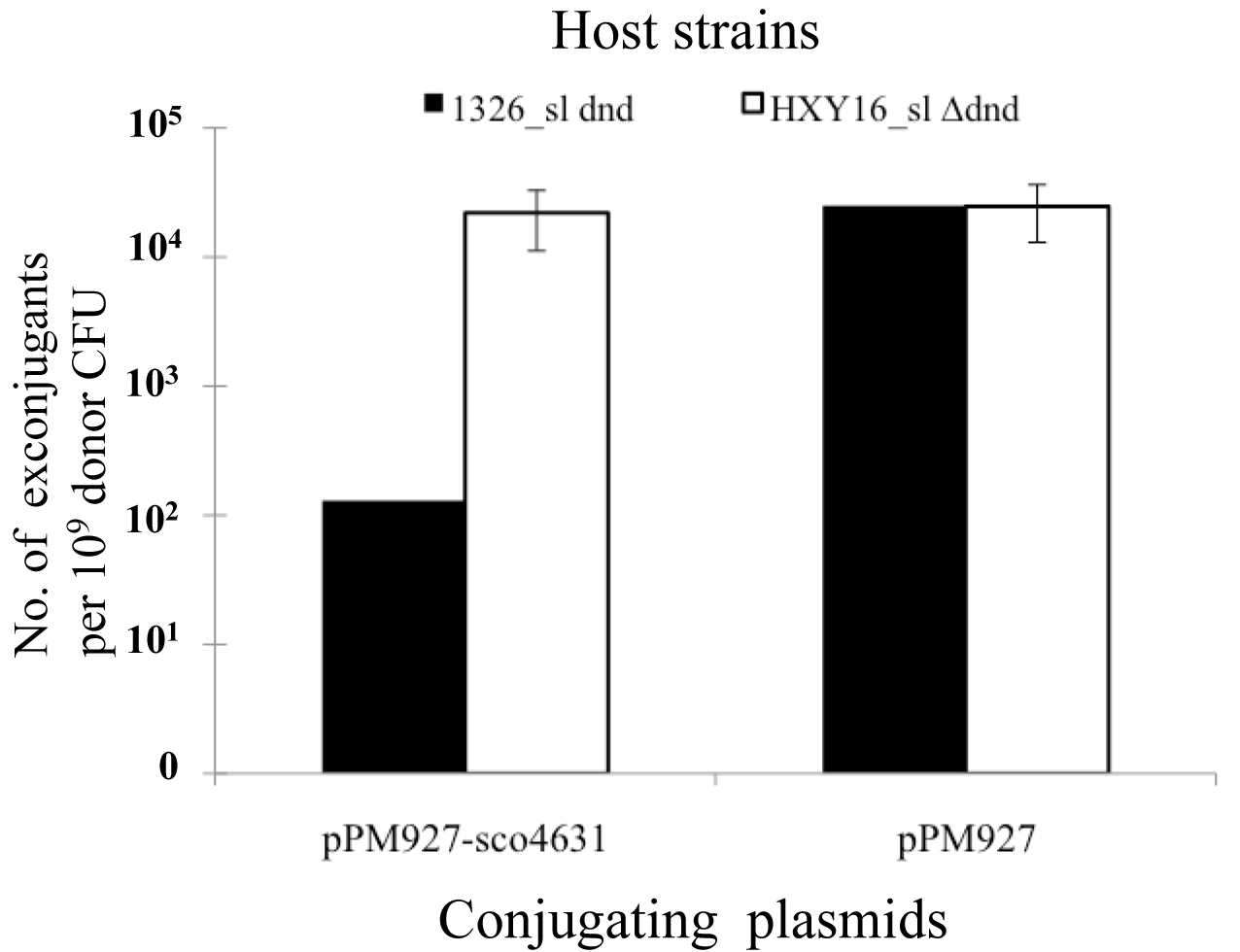

Supplement: Figure S4 — Transfer of sco4631 from E. coli to S. lividans strains. HXY16 is the S. lividans derivative which lacks the dnd gene cluster; pPM927-sco4631 (pJTU1654) is pPM927 harboring sco4631 with its native promoter. E. coli strain used for conjugation is ET12567::pUZ8002. (0.15 MB TIF) [file pgen.1001253.s004.tif]

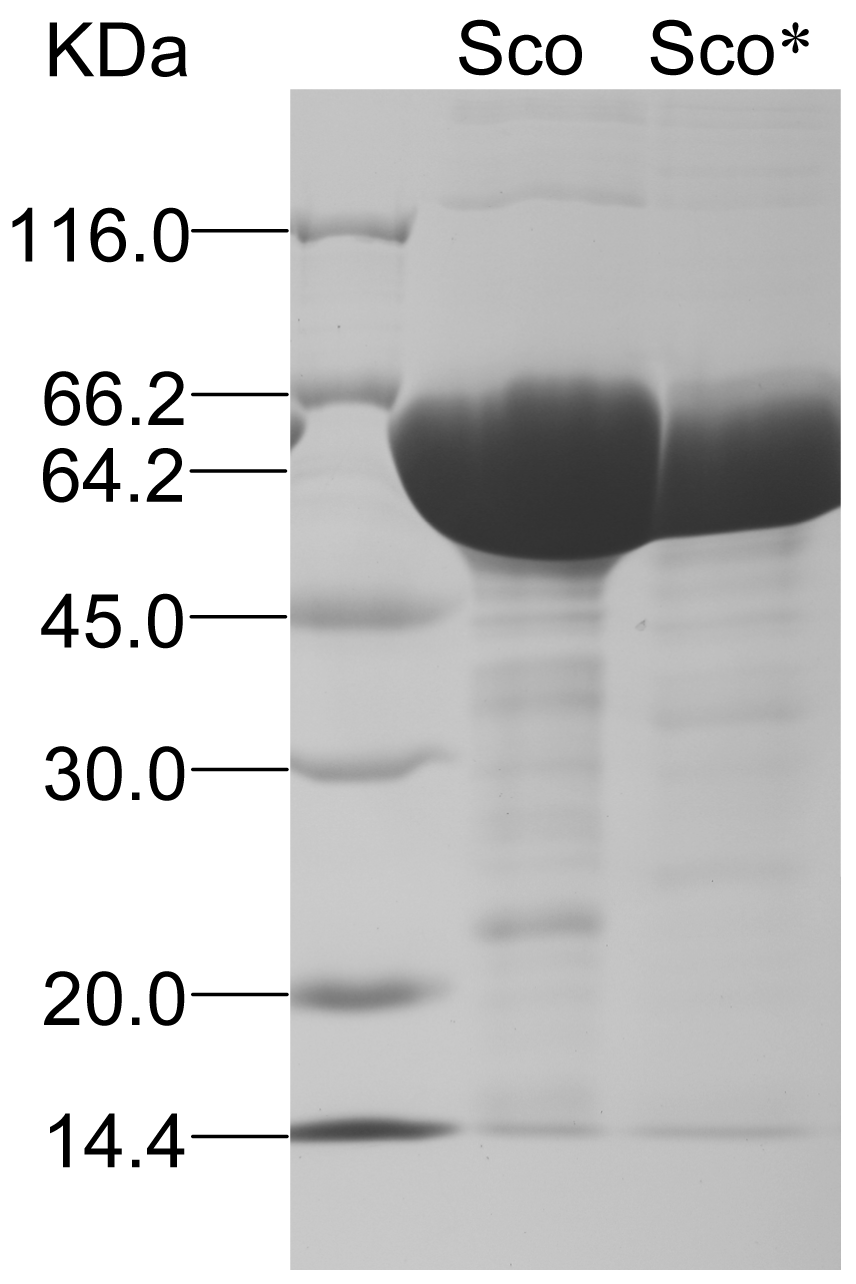

Supplement: Figure S5 — Heterologous expression of His6-tagged Sco4631 and its mutant in E. coli. Sco, protein eluted from the Ni affinity column as a 64.2 kd polypeptide. M, size markers. (0.67 MB TIF) [file pgen.1001253.s005.tif]

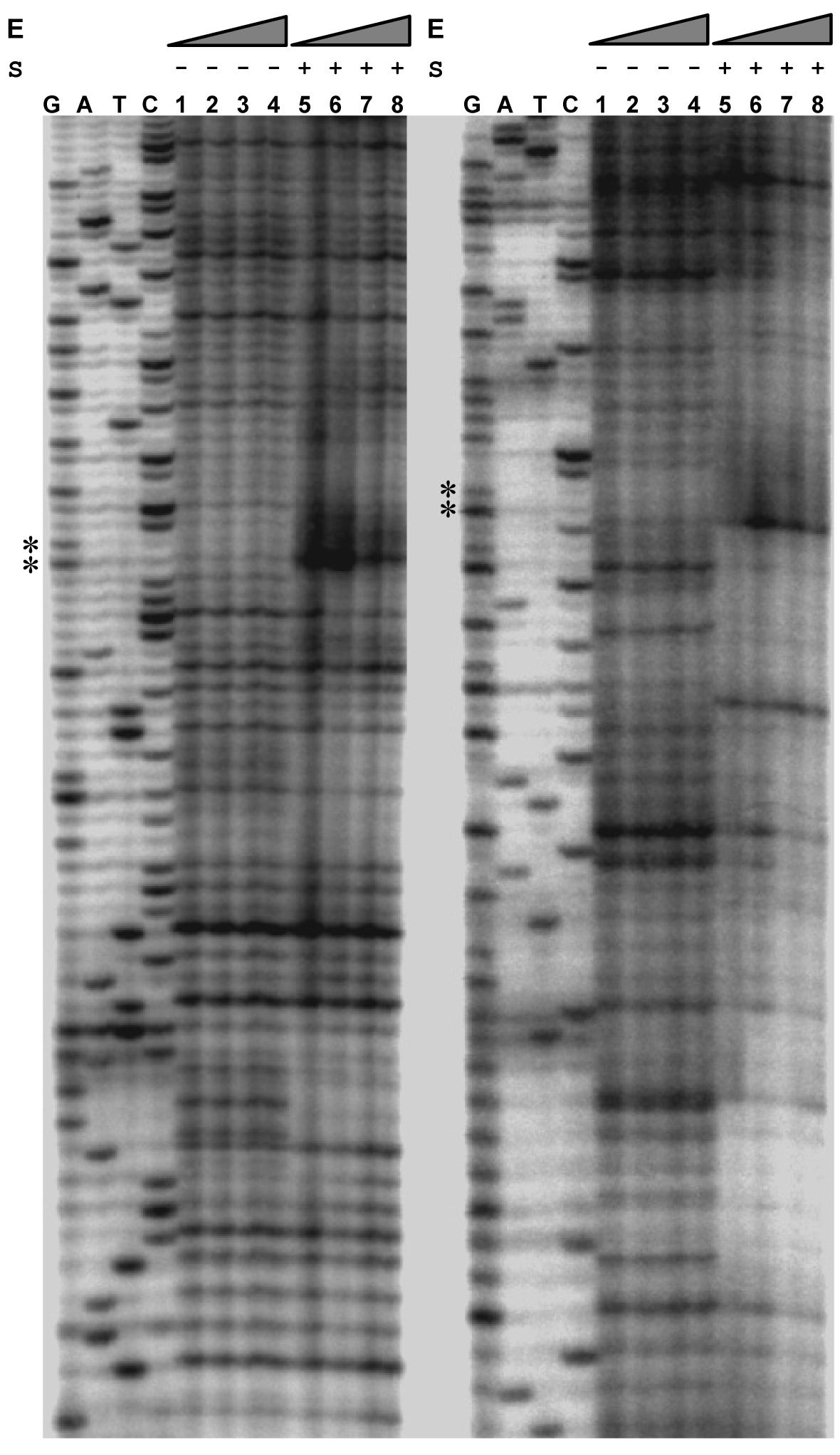

Supplement: Figure S6 — DNase I protection assay of a synthetic S-modified (phosphorothioated) double-stranded 118 bp oligonucleotide by purified His6-Sco4631. Autoradiographs of 5′ labeled top strand DNA (left) and 5′ labeled bottom strand DNA (right). G, A, T, C denote the sequencing ladders. ** indicates the tandem G residues linked by a phosphorothioate bond. Lanes 1–4 are controls containing unphosphorothioated DNA and increasing amounts of active His6-Sco4631. Lanes 5–8 contained phosphorothioated DNA and increasing amounts of the active His6-Sco4631. Lanes 1 and 5 contained no enzyme, and lanes 2–4 and lanes 6–8 contained 1.1, 4.5 and 18 µM enzyme, respectively. (3.20 MB TIF) [file pgen.1001253.s006.tif]

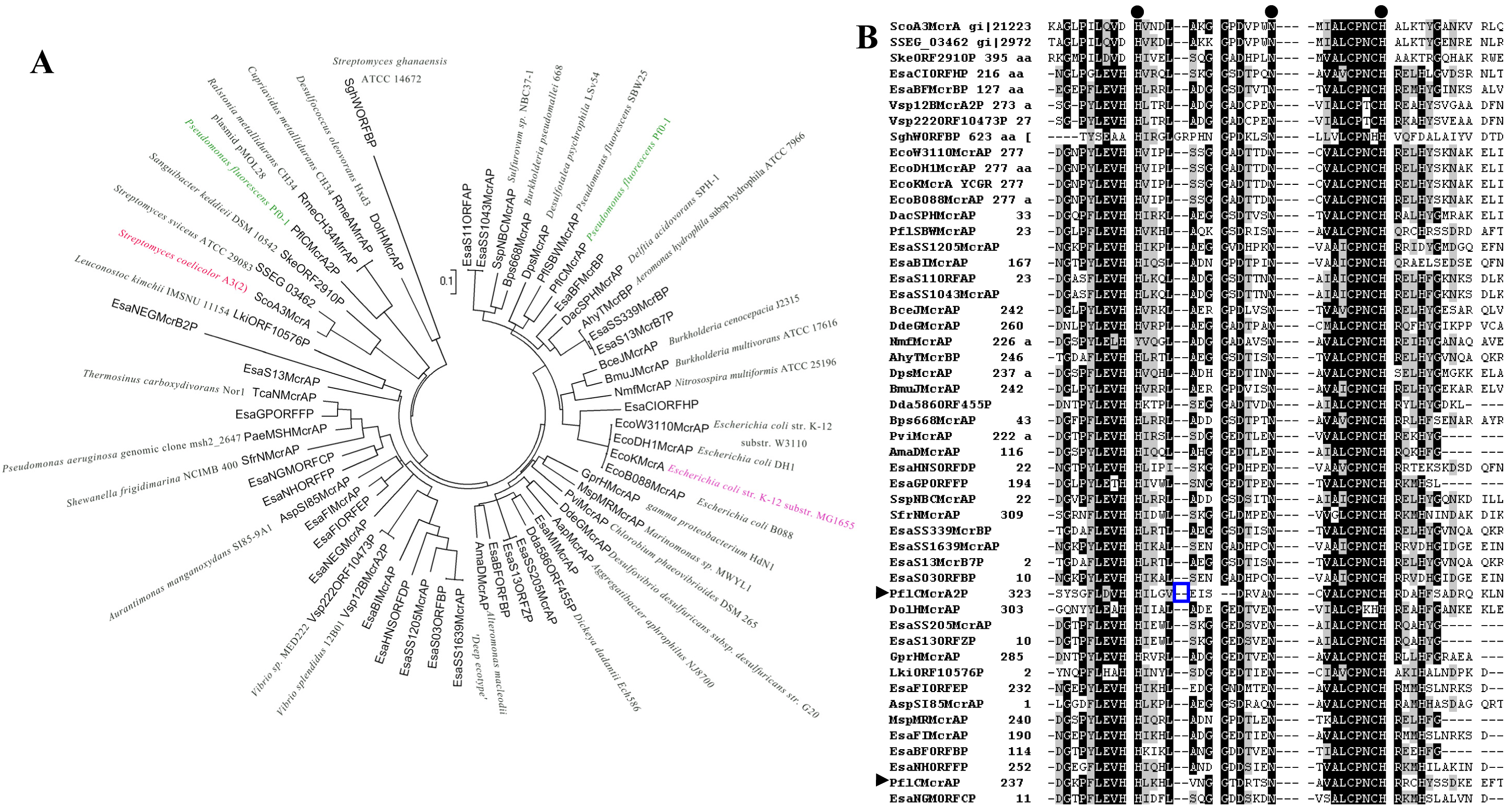

Supplement: Figure S7 — Phylogenetic analysis of Sco4631. A. Unrooted phylogenetic tree showing the extreme diversity of Sco4631 and its closest homologues. The 560 aa sequence of Sco4631 was used as a template to search REbase and the non-redundant protein database using BlastP for Type IV REases. The 59 top scoring sequences (E<10−5) were aligned using ClustalW. The scale represents an evolutionary distance of 0.1. Red, ScoA3McrA = Sco4631 is most similar to the 481 aa conserved hypothetical protein SSEG03462 of Streptomyces sviceus ATCC 29083 (389/466 = 83% identity) a producer of many oxidative antibiotic tailoring enzymes. The next closest homologue is the putative HNH endonuclease SkeORF2910P from the Actinomycete Sanguibacter keddieii DSM 10542 which was isolated from bovine blood. Purple, EcoKMcrA from the e14 prophage in E. coli K-12, and from three other E. coli strains containing the e14 prophage. EcoKMcrA has an evolutionary distance >0.8 from ScoA3McrA, and only 34/91 aa identity in the HNH region. Green, two putative HNH endonucleases from Pseudomonas fluorescens Pf0-1 which contains a complete dnd gene cluster and S-modified (phosphorothioated) DNA. This strain was thus not expected to contain a protein that cleaves S-modified DNA. The evolutionary distance of these proteins is about 1, and the identity is only about 40% in a 65 aa region containing the HNH conserved sequence motif. One of the proteins (McrA2P) may be inactive because it lacks two highly conserved amino acids (GE) in the centre of the HNH motif (Hx13Nx8H; see Figure McrA HNH alignments). Note, 22 of the 59 Esa proteins are from environmental DNA samples. B. Mutiple alignment of ScoA3McrA and the 58 most similar proteins. The dots above the sequence mark H, N and H of the conserved Hx13Nx8H (HNH) motif. Arrows to the left mark sequences from Pseudomonas fluorescens Pf0-1 which contains S-modified DNA. The deletion of two amino acid residues in PflCMcrA2P are marked with blue rectangle. (2.00 MB TIF) [file pgen.1001253.s007.tif]
