# Supplementary material for: Cleavage of Phosphorothioated DNA and Methylated DNA by the Type IV Restriction Endonuclease ScoMcrA
Source: PLoS Genet. 2010 Dec 23;6(12):e1001253. doi: 10.1371/journal.pgen.1001253 (PMC3009677; doi:10.1371/journal.pgen.1001253)
Supplement: Table S1 — Strains and plasmids used in this study. (0.06 MB DOC) [file pgen.1001253.s008.doc]

**TableS1: Strains and plasmids used in this study**

| ***S. coelicolor*** | **Relevant properties** | **Source or reference** |
| --- | --- | --- |
| M145 | *Streptomyces coelicolor* wild -type strain, SCP1^-^ SCP2^-^ | [1] |
| LG3 | M145 derivative harbouring a deletion of *sco4631-*bearing GI-11 | This study |
| LG4 | M145 derivative harbouring a pJTU1653-interrupted *sco463*1 | This study |
| ***S. lividans*** |  |  |
| *S. lividans 66* | Wild type strain | [1] |
| HXY6 | *S. lividans* 1326 derivative with a *dnd* gene cluster deletion | [2] |
| HXY16 | *S. lividans* 1326 derivative with a precise deletion of SLG | [2] |
| LG5 | HXY16 containing pJTU1654 | This study |
| LG6 | HXY16 containing pPM927 | This study |
| ***Escherichia coli*** |  |  |
| DH10B | F^-^ *mcr*A ∆(*mrr- hsd*RMS- *mcr*BC) φ80*lac*Z∆M15 ∆*lac*X74 *rec*A1 *end*A1 *ara*D139 ∆(*ara, leu*)7697 *gal*U *gal*K λ^-^ *rps*L *nup*G | GIBCO BRL |
| BL21(DE3)pLysE | F^-^ *ompT* *hsdS_b_* (*r_b_^-^ (2)m_b_^-^* ) *gal dcm* (DE3) pLysE | Novagen |
| ET12567  /pUZ8002 | Strain used for conjugation between *E. coli* and *Streptomyces* spp. *rec*F, *dam*, *dcm*, *hsdS,* Cml^r^, Km^r^, pUZ8002 | [3] |
| ET12567 | *dcm*, *hsdS* Cml^r^, | [3] |
| **Plasmid** |  |  |
| pJTU412 | Highly unstable replicative vector*, tsr, bla, sti^−^, rep*, orf85^−^, R1^−^* | [4] |
| pSET151 | Suicide vector for *Streptomyces*. *bla, oriT, tsr, lacZ,* | [5] |
| pSET152 | *aac(3)IV, lacZ*, *rep*^pUC^*, att* ^φC31^*, int* ^φC31^*, oriT* | [5] |
| pPM927 | *aadA, tsr, att* ^pSAM2^*, int* ^pSAM2^ | [6] |
| pHZ1904 | pSET152 derivative containing *dnd* | [7] |
| pHZ1904* | pHZ1904 mutant with a point *dndE* mutation | This study |
| pHZ209 | pIJ101 derivative, *tsr, oriT*, *rep*^pIJ101^ | [8] |
| pJTU1651 | pJTU412 containing *dnd* gene cluster | This study |
| pJTU1652 | pBluescript derivative containing 1483 bp internal region of *sco4631* | This study |
| pJTU1653 | pSET151 containing 1204 bp internal region of *sco4631* | This study |
| pJTU1654 | pPM927 harboring *sco4631* with its native promoter | This study |
| pJTU1655 | pET28a derivative for heterologous expression and purification of SCO4631 | This study |
| pSco4631H508A | a mutant of pJTU1655 with the product of H508A | This study |

**References – Supporting Information**

1. Kieser T, Bibb MJ, Buttner MJ, Chater KF, Hopwood DA (2000) Practical *Streptomyces* genetics. Norwich: The John Innes Foundation.

2. He X, Ou HY, Yu Q, Zhou X, Wu J, et al. (2007) Analysis of a genomic island housing genes for DNA S-modification system in Streptomyces lividans 66 and its counterparts in other distantly related bacteria. Mol Microbiol 65: 1034-1048.

3. Flett F, Mersinias V, Smith CP (1997) High efficiency intergeneric conjugal transfer of plasmid DNA from Escherichia coli to methyl DNA-restricting streptomycetes. FEMS Microbiol Lett 155: 223-229.

4. Sun Y, He X, Liang J, Zhou X, Deng Z (2008) Analysis of functions in plasmid pHZ1358 influencing its genetic and structural stability in Streptomyces lividans 1326. Appl Microbiol Biotechnol.

5. Bierman M, Logan R, O'Brien K, Seno ET, Rao RN, et al. (1992) Plasmid cloning vectors for the conjugal transfer of DNA from Escherichia coli to Streptomyces spp. Gene 116: 43-49.

6. Smokvina T, Mazodier P, Boccard F, Thompson CJ, Guerineau M (1990) Construction of a series of pSAM2-based integrative vectors for use in actinomycetes. Gene 94: 53-59.

7. Zhou X, He X, Liang J, Li A, Xu T, et al. (2005) A novel DNA modification by sulphur. Mol Microbiol 57: 1428-1438.

8. Liang J, Wang Z, He X, Li J, Zhou X, et al. (2007) DNA modification by sulfur: analysis of the sequence recognition specificity surrounding the modification sites. Nucleic Acids Res 35: 2944-2954.
