# Supplementary material for: Cleavage of Phosphorothioated DNA and Methylated DNA by the Type IV Restriction Endonuclease ScoMcrA
Source: PLoS Genet. 2010 Dec 23;6(12):e1001253. doi: 10.1371/journal.pgen.1001253 (PMC3009677; doi:10.1371/journal.pgen.1001253)
Supplement: Table S2 — Primers used in this study. (0.05 MB DOC) [file pgen.1001253.s009.doc]

| **Primer** | **Primer sequence** | Reference |
| --- | --- | --- |
| S31DF | 5’-CTTGAGCACGAAGGAAGGC-3’ | This study |
| S31DR | 5’-TGAGGGCATGGCAGTTGG-3’ | This study |
| S31HEF | 5’-CGTCTAGACGCCTACTCTTG-3’ | This study |
| S31HER | 5’- CAGTCTAGAATGCGGGTTAC-3’ | This study |
| tsrtestF | 5’-TTGGACACCATCGCAAATC- 3’ | This study |
| tsrtestR | 5- CGGGAAGGGAGAAGACGT-3’ | This study |
| S31OEF | 5’- CATATGGCACCTTCGGAGAT-3’ | This study |
| S31OER | 5’- GGGCTCAGGCAGCGTAAT-3’ | This study |
| H508A-F | 5’- TGCAGGTTGACGCCGTCAAT-3’ | This study |
| H508A-R | 5’- GGATGGGCAGCCCAGCCTTG -3’ | This study |
| MF | 5’-TAAGCTTAACGGCGTCAGCCGGGCAGGAT  AGGTGAAGTAGGCCCACCCGCGAGCGGGTG  TTCCTTCTTCACTGTCCCTTATTCGCAC ^m5^**C**T-3’ | This study |
| MR | 5’-GCCGAATTCCAGCAGGTAGGCCGACAGGC  TCATGCCGGCCAGCCTCGCAGAGCAGGATTC  CCGTTGAGCACCGC ^m5^**C**A-3’ | This study |
| UMF | 5’-TAAGCTTAACGGCGTCAG-3’ | This study |
| UMR | 5’-GCCGAATTCCAGCAGGTA-3’ | This study |
| SF | 5’-CCGGATCCGCCGACCCCGGGCGAGTAATC  CCAGGATTACTCCCGCGGCTTCGACCCCGpsG-3’ | This study |
| SR | 5’-CGCAGCTGGTCATCCCGGTGACGTACGGCG  GGGGTCGGTGACGTACGCGGCGACGGCGpsG-3’ | This study |
| USF | 5’- CCGGATCCGCCGACCCCGGG-3’ | This study |
| USR | 5’- CGCAGCTGGTCATCCCGGTG-3’ | This study |
| Lp1F | 5’-ATGGCGTTCACGGACTGG-3’ | [2] |
| Rp1R | 5’-CATCCACGACTGGGTCTACG-3’ | [2] |
| GITestF | 5’-CAGTGCCTTGAGCGAGATGC-3’ | This study |
| GITestR | 5’-GGTCGGCGAGTTCGGTGTAG-3’ | This study |
| seqF | 5’-AAAGGGAATAAGGGCGACAC-3’ | This study |
| seqR | 5’-CAGGCAACTATGGATGAACG-3’ | This study |

**Table S2: Primers used in this study**
